# Genetic risk factors have a substantial impact on healthy life years

**DOI:** 10.1038/s41591-022-01957-2

**Published:** 2022-09-12

**Authors:** Sakari Jukarainen, Tuomo Kiiskinen, Sara Kuitunen, Aki S. Havulinna, Juha Karjalainen, Mattia Cordioli, Joel T. Rämö, Nina Mars, FinnGen, Kaitlin E. Samocha, Hanna M. Ollila, Matti Pirinen, Andrea Ganna

**Affiliations:** 1 Institute for Molecular Medicine Finland (FIMM), University of Helsinki, Helsinki, Finland; 2 Finnish Institute for Health and Welfare, Helsinki, Finland; 3 Program in Medical and Population Genetics, Broad Institute of MIT and Harvard, Cambridge, MA USA; 4 Analytic and Translational Genetics Unit, Massachusetts General Hospital, Boston, MA USA; 5 Center for Genomic Medicine, Massachusetts General Hospital, Boston, MA USA; 6 Anesthesia, Critical Care and Pain Medicine, Massachusetts General Hospital and Harvard Medical School, Boston, MA USA; 7 Department of Public Health, University of Helsinki, Helsinki, Finland; 8 Helsinki Institute for Information Technology HIIT and Department of Mathematics and Statistics, University of Helsinki, Helsinki, Finland

**Keywords:** Genetics, Epidemiology, Public health, Genetics research, Risk factors

## Abstract

The impact of genetic variation on overall disease burden has not been comprehensively evaluated. We introduce an approach to estimate the effect of genetic risk factors on disability-adjusted life years (DALYs; ‘lost healthy life years’). We use genetic information from 735,748 individuals and consider 80 diseases. Rare variants had the highest effect on DALYs at the individual level. Among common variants, rs3798220 (*LPA*) had the strongest individual-level effect, with 1.18 DALYs from carrying 1 versus 0 copies. Being in the top 10% versus the bottom 90% of a polygenic score for multisite chronic pain had an effect of 3.63 DALYs. Some common variants had a population-level effect comparable to modifiable risk factors such as high sodium intake and low physical activity. Attributable DALYs vary between males and females for some genetic exposures. Genetic risk factors can explain a sizable number of healthy life years lost both at the individual and population level.

## Abstract (web summary)

A new analysis combining data from large biobanks and the Global Burden of Disease study estimates that genetic risk factors significantly impact the number of healthy life years lost both at the individual and population level

## Main

Genome-wide association studies (GWASs) have identified thousands of variants associated with biological traits and diseases^1^. Overall, these results demonstrate widespread pleiotropy (genetic variants associated with more than one trait)^2^. Studies commonly quantify the impact of genetic variation on a single disease at a time^3–5^, or when considering multiple diseases^6,7^, do not use a single metric that can capture overall disease burden, apart from studies on lifespan^8–10^. It is therefore challenging to assess the impact of genetic variation on overall health and to compare the total impact of different variants.

Past efforts in comparative risk assessment involve quantifying the effects of modifiable exposures (for example, sodium intake) on health outcomes to inform public health measures^11^. This type of assessment has not been systematically performed for genetic risk factors. The utility of genetic information for rare disease diagnosis^12^ and inherited cancer syndromes^13^ is well established. In contrast, despite major advances and ongoing research investments, the viability, utility and cost-effectiveness of other applications such as genetic screening for common diseases^14^, risk stratification via polygenic scores (PGSs)^15–18^, in vivo gene editing^19–21^ and, most controversially, embryo selection^22–26^ remain uncertain. A comparative risk assessment framework for genetic risk factors can help to develop and evaluate these attempts to make genetic information actionable.

**Extended Data Fig. 1:**
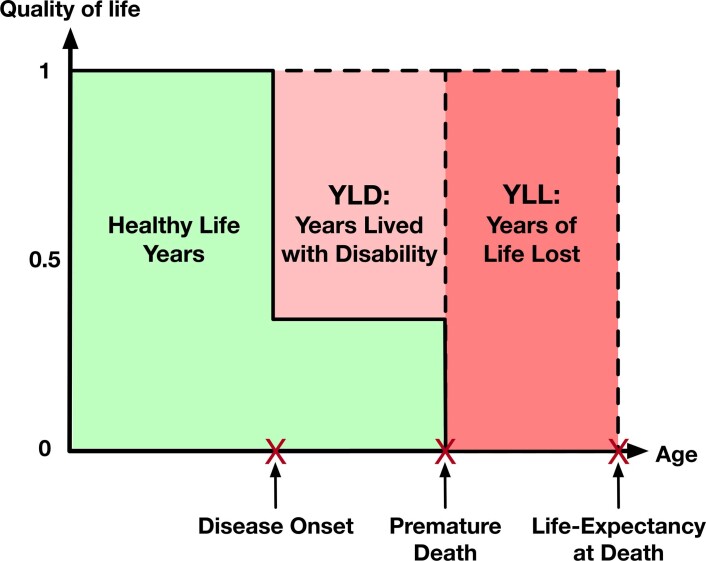
Schematic representation of how disability-adjusted life years (DALYs) are constructed from years lived with disability (YLD), and years of life lost (YLL). DALYs are a metric for measuring population-level disease burden that combines a measure of premature mortality called years of life lost (YLL) and a measure of healthy life years lost due to lowered quality of life called years lived with disability (YLD), so DALYs are the sum of YLDs and YLLs.

One prominent metric for disease burden is the DALY. DALYs represent the loss of healthy life years through worsened quality of life and premature death attributable to a disease^27^. Combining both quality of life and mortality into a single metric, DALYs are used to monitor disease burden across hundreds of countries in the Global Burden of Disease (GBD) study^11,27^. GBD estimates the yearly amount of DALYs in each country that are attributable to a list of collectively exhaustive and non-overlapping diseases and injuries^27^. DALYs are the sum of years lived with disability (YLDs; ‘lowered quality of life’) and years of life lost (YLLs; ‘premature death’) (Extended Data Fig. 1).

We present an approach for combining genetic association results for 80 diseases from two biobank studies with DALY estimates from the 2019 GBD study^27^ to provide an overview of the impact of genetic variation on lost healthy life years both at an individual and population level. We rank different genetic risk factors in terms of their health impact and compare genetic risk factors with traditional modifiable risk factors, presenting a template for comparative risk assessment of genetic risk factors.

## Results

### Estimating attributable DALYs

**Fig. 1:**
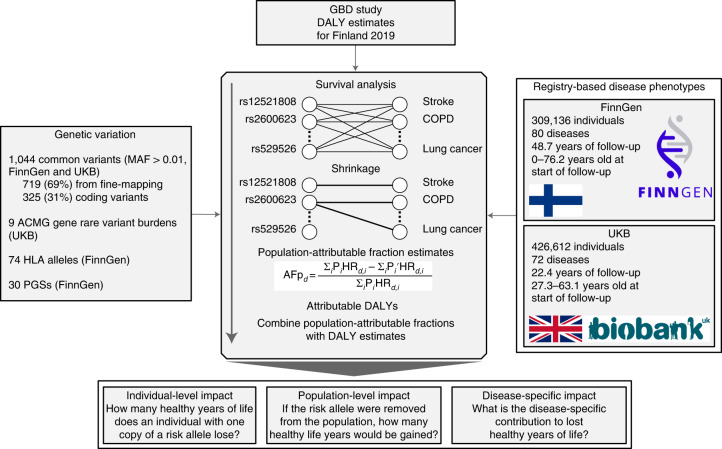
Study overview. AFp, population-attributable fraction.

Our method is similar to the GBD approach, which estimates the disease burden attributable to modifiable risk factors^11^, except here we consider different classes of genetic risk factors: common variants, rare deleterious variants, human leukocyte antigen (HLA) alleles, *APOE* haplotypes and PGSs (Fig. 1). To estimate genetic associations, we used individual-level data from two biobank studies: FinnGen^28^ (*n* = 309,136) and UK Biobank^29^ (UKB; *n* = 426,612) with registry-based follow-up of 48.7 and 22.4 years, respectively (Supplementary Table 1). In total, we considered 80 non-communicable diseases that account for 83.1% of the total DALYs out of all non-communicable diseases in Finland 2019 (ref. ^27^) (Supplementary Tables 2 and 3).

For each genetic exposure–disease pair, we estimated the hazard ratio (HR) using a Cox proportional hazards model. Because a single genetic variant is expected to affect only a minority of the considered 80 diseases, we used a shrinkage approach with a spike-and-slab type prior distribution for the log HRs of each genetic exposure for the 80 diseases. We discarded any HRs where the posterior probability of the null model was above 10%.

**Extended Data Fig. 2:**
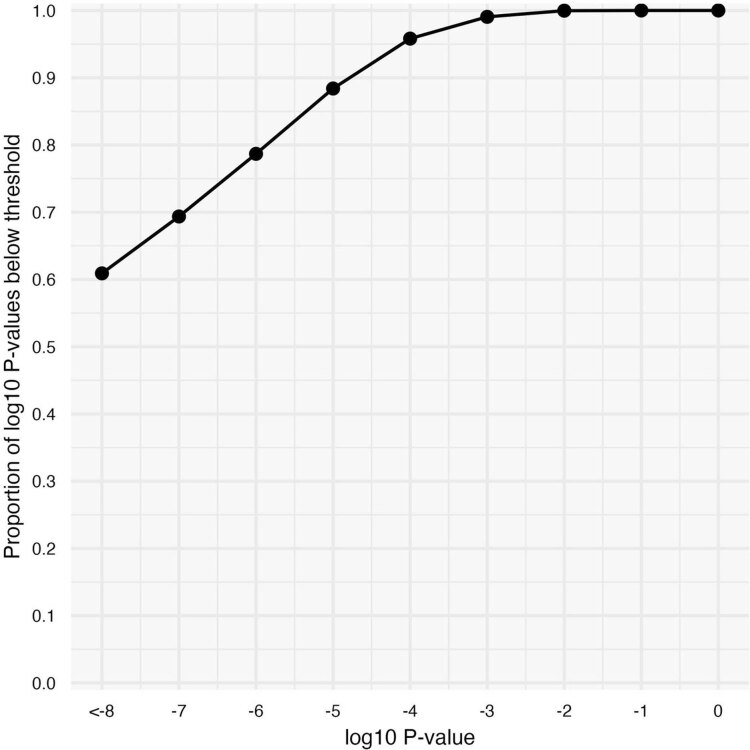
Cumulative distribution plot of log_10_*P*-values for all 3,123 HRs between genetic exposures and diseases. 67.1% of the associations were genome-wide significant (*P* < 5×10^−8^) and 99% had an association with *P* < 7.3×10^−4^. Estimates are based on 735,748 individuals. *P*-values are for two-sided Wald tests.

Overall, we estimated the HRs through 92,560 survival analyses: associations of 1,044 common variants, 9 rare variant gene burdens, 74 HLA alleles and 30 PGSs with 80 diseases. After shrinkage, we retained 3,123 HRs for genetic exposure–disease pairs, most of which (67.1%) were genome-wide significant (*P* < 5 × 10^−8^) and 99% had an association with *P* < 7.3 × 10^−4^ (Extended Data Fig. 2). Using the HR estimates and frequencies of the genetic exposures, we estimated the population-attributable fraction of disease cases for each genetic exposure (proportion of cases prevented if the exposure was removed). We combined attributable fractions with disease-specific population DALYs for Finland 2019 from GBD^27^ (Supplementary Table 3) to obtain attributable DALY estimates. Finally, for each genetic exposure, we summed attributable DALYs across the 80 diseases to estimate the total impact. The individual-attributable DALYs, our main measure of interest, can roughly be interpreted as the expected lost healthy life years for an individual attributable to the genetic exposure.

### Attributable DALYs for common variants

**Extended Data Fig. 3:**
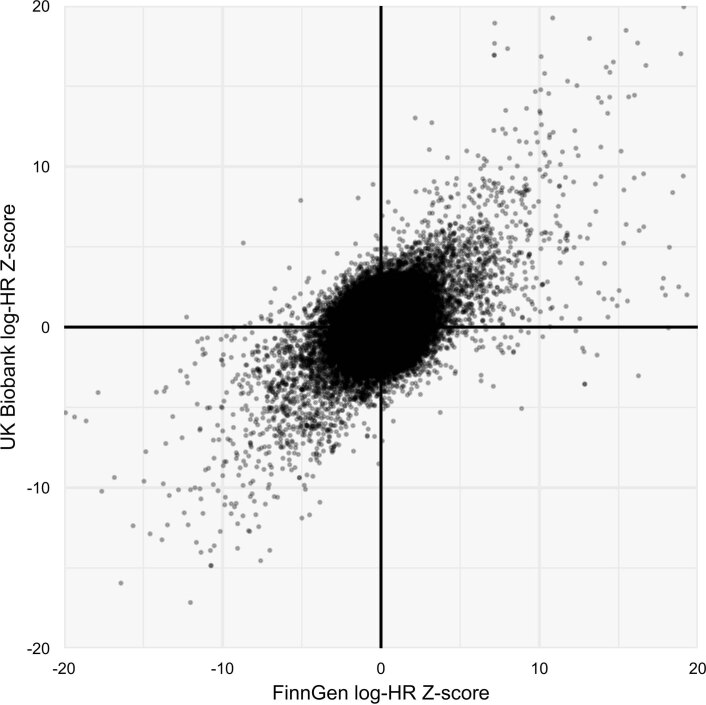
Comparison of effect sizes (log-HR *Z*-score) for common variant-disease associations (n = 68,616) estimated both in FinnGen (n = 309,136) and UK Biobank (n = 426,612). Axes are truncated at ±20, leaving out 36 points.

We considered 1,044 independent common variants with minor allele frequency (MAF) over 0.01 (Supplementary Table 4). We selected 564 of these based on having at least one *P* < 5 × 10^−8^ association with any of the 80 diseases and having the highest probability of being causal within a sum-of-single-effects (SuSiE) fine-mapped^30^ 95% credible set in FinnGen. Additionally, we selected 155 common variants with at least one *P* < 5 × 10^−12^ association with six traditional risk factor traits (body mass index (BMI), glycated hemoglobin (HbA1c), high-density lipoprotein (HDL) cholesterol, low-density lipoprotein (LDL) cholesterol, systolic blood pressure and cigarettes per day) that had the highest probability of being causal within a SuSiE fine-mapped 95% credible set in UKB^31^. Last, we included 325 coding variants having a *P* < 5 × 10^−8^ association with one of the diseases in FinnGen. Among the 1,044 variants, 34.6% were annotated as missense (*n* = 335) or putative loss-of-function (pLOF; *n* = 26). The HRs for common variants were comparable between FinnGen and UKB (Extended Data Fig. 3) and we consequently meta-analyzed the HRs. All estimates are for the comparison of one versus zero copies of the minor allele, so the individual-attributable DALYs correspond to the expected loss of healthy life years if an individual with no copies of the minor allele had instead one copy at birth.

**Fig. 2:**
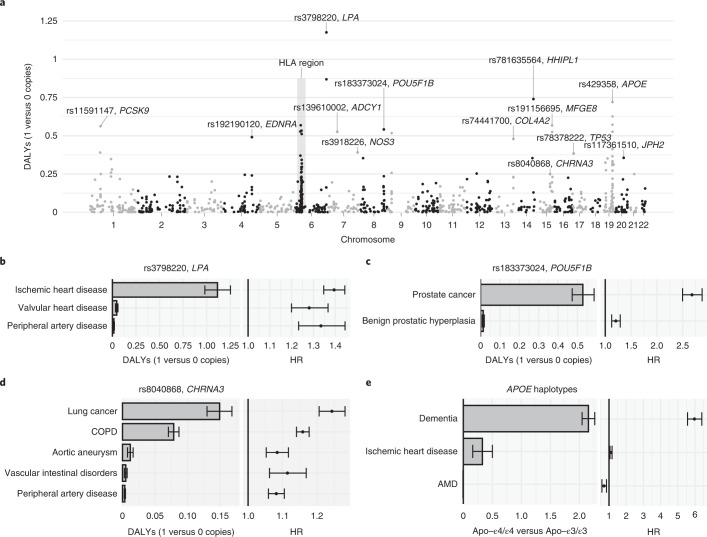
Effect of common variants on DALYs. **a**, Absolute effect on DALYs from carrying one versus zero copies of the minor allele for each common variant. We separately analyzed imputed alleles in the HLA region (Extended Data Fig. 4). **b**–**e**, For three common variants and the *APOE* haplotypes, we reported attributable DALYs and HRs separately for each disease. Estimates are based on 735,748 individuals (*n* = 309,136 for **e**). Error bars denote 95% CIs. AMD, age-related macular degeneration.

**Extended Data Fig. 4:**
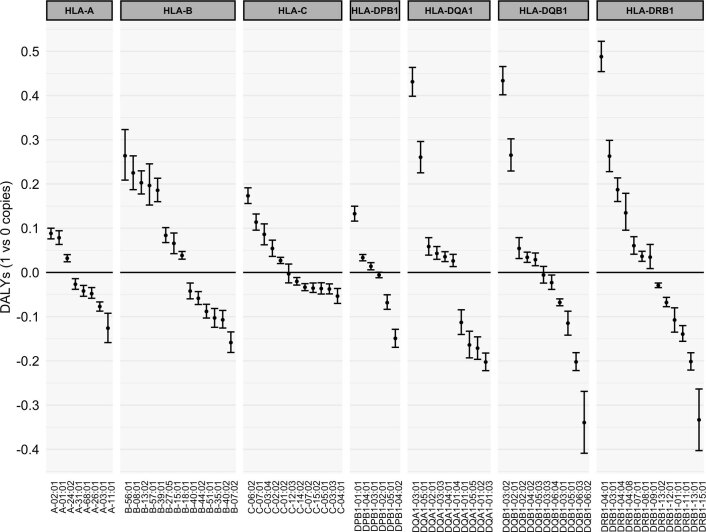
Effect of HLA alleles on DALYs. Note that each gene is multiallelic, so the comparison of ‘1 vs 0 copies’ is more precisely the effect for having ‘1 copy of the allele in question and 1 average copy of other allele’ vs ‘having 0 copies of the allele in question and 2 average copies other alleles’. The interpretation of attributable DALYs in this context is not straightforward since these represent multiallelic loci. Estimates are based on 309,136 individuals (FinnGen). Error bars denote 95% confidence intervals.

Overall, carrying one versus zero copies of the common variants resulted in relatively small effects on lost healthy life years (DALYs), with only 56 out of 1,044 (5.4%) variants with over 0.25 attributable DALYs (Supplementary Tables 5 and 6). Many of the top variants were in chromosome 6, both inside and outside the HLA region (Fig. 2a). We provide attributable DALYs for HLA alleles in Extended Data Fig. 4.

The variant with the highest number of attributable DALYs was rs3798220, a missense variant in *LPA*, with 1.18 (95% confidence interval (CI) 1.03–1.32) attributable DALYs from carrying one versus zero copies of the C allele (Fig. 2b). The effect was almost exclusively through ischemic heart disease (1.11 DALYs) and to a lesser extent through non-rheumatic valvular heart disease (0.046 DALYs) and lower extremity peripheral artery disease (0.016 DALYs) despite similar relative risk increases. This is because of the larger number of population DALYs attributed to ischemic heart disease by the GBD (60-fold difference to lower extremity peripheral artery disease; Supplementary Table 3), highlighting the relevance of considering absolute measures of disease burden.

One notable example is rs183373024, a noncoding variant near the *POU5F1B* gene^32^, with 0.54 (CI 0.48–0.60) attributable DALYs, mainly due to prostate cancer (Fig. 2c). Another example is rs8040868, a synonymous variant in the well-known *CHRNA5/A3/B4* gene cluster associated with nicotine dependence^33^, with 0.25 (CI 0.23–0.27) attributable DALYs (Fig. 2d), with effects through lung cancer, chronic obstructive pulmonary disease (COPD), aortic aneurysm, vascular intestinal disorders and lower extremity peripheral artery disease (all consequences of smoking).

Given the strong associations between *APOE* alleles and longevity^34^, we defined the three main *APOE* alleles determined by rs429358 and rs7412. Carrying the most deleterious Apo-ε4/ε4 haplotype instead of the most common Apo-ε3/ε3 resulted in 2.48 (CI 2.28–2.68) attributable DALYs, mainly through an increase in the risk of Alzheimer’s disease and other dementias (HR = 5.97, CI 5.57–6.40; Fig. 2e). Overall, out of the top 10% common variants with the highest number of attributable DALYs, 49.4% were significantly associated (*P* < 0.05) with longevity in the largest GWAS on lifespan^10^ versus 18% in the bottom 10%.

### Attributable DALYs for rare deleterious variants

We used whole-exome sequencing data from UKB (*n* = 174,379) to estimate attributable DALYs for rare deleterious coding variants (MAF < 0.001). The American College of Medical Genetics and Genomics (ACMG) recommends reporting incidental findings in clinical exome and genome sequencing for 73 genes^35,36^. We estimated the attributable DALYs for two types of burdens for these ACMG genes: (1) putative loss-of-function (pLOF) variant burden and (2) ‘pathogenic’ or ‘likely pathogenic’ Clinvar^37^ variant burden (for *BRCA1*/*2* we used ‘pathogenic’ ENIGMA^38^ variants instead). We report results for nine genes with at least 35 individuals with a positive burden and at least one disease association.

**Fig. 3:**
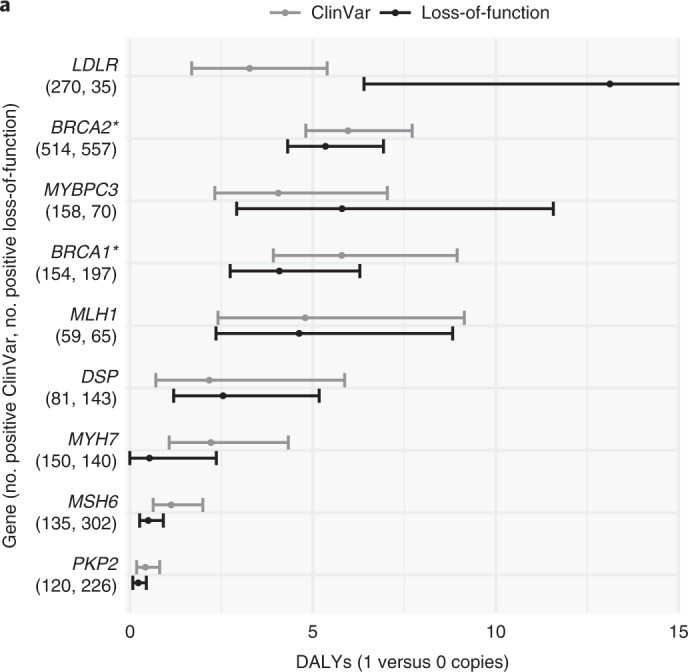
DALYs attributable to carrying a deleterious rare variant in ACMG genes. The ClinVar^37^ burden contains all variants annotated as ‘pathogenic’ or ‘likely pathogenic’. *For *BRCA1* and *BRCA2* we only considered variants from ENIGMA^38^ annotated as ‘pathogenic’. The loss-of-function burden contains all variants annotated as pLOF with high confidence^60^. Estimates are based on 174,379 individuals from UKB. Error bars denote 95% CIs.

The five most impactful genes (Fig. 3 and Supplementary Tables 7–9) were *LDLR* (ischemic heart disease), *BRCA2* (breast, ovarian, liver and prostate cancer, and COPD), *MYBPC3* (cardiomyopathy and myocarditis), *BRCA1* (breast and ovarian cancer) and *MLH1* (colon and rectum cancer). As an example, individuals carrying one pLOF in *BRCA1* lose on average 4.08 (CI 2.74–6.32, *P* = 1.4 × 10^−5^) healthy life years through breast cancer (2.11 DALYs, CI 1.39–3.14; HR = 7.01, CI 4.94–9.94) and ovarian cancer (1.97 DALYs, CI 0.95–3.93; HR = 16.2, CI 8.22–31.8).

### Attributable DALYs for polygenic scores

**Fig. 4:**
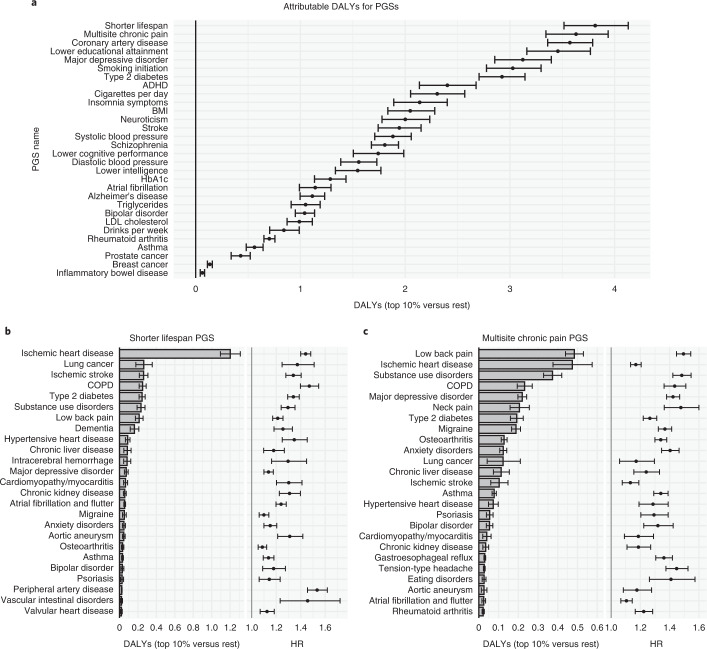
Polygenic score impact on DALYs. **a**, DALYs attributable to belonging in the top 10% versus bottom 90% of each PGS. **b**,**c**, Top 25 diseases in terms of attributable DALYs and HRs for two PGSs (bold in **a**). Estimates are based on 309,136 individuals (FinnGen). Error bars denote 95% CIs. ADHD, attention deficit hyperactivity disorder.

We considered 30 PGSs (Supplementary Table 10) covering major diseases, risk factors and psychobehavioral traits in a FinnGen-only analysis. We estimated individual-attributable DALYs as the expected loss of healthy life years if an individual in the bottom 90% of a PGS was instead in the top 10% at birth. Overall, the attributable DALYs varied from 0.07 (inflammatory bowel disease^39^) to 3.81 (shorter lifespan^10^) (Fig. 4a and Supplementary Tables 11 and 12). Many of the PGSs exhibited substantial pleiotropy, with a median of 16 (interquartile range (IQR) 9–28) PGS-disease associations remaining after shrinkage.

The shorter lifespan^10^ PGS had the highest impact. Individuals in the top 10% of the PGS are expected to lose 3.81 (CI 3.52–4.13) healthy life years compared to an individual in the bottom 90% (Fig. 4b). This PGS acts mainly through ischemic heart disease (1.2 DALYs) and to a lesser extent through lung cancer, ischemic stroke, COPD, type 2 diabetes, substance use disorders and low back pain (0.21–0.26 DALYs each). Notably, the PGS for multisite chronic pain^40^ had the second-highest impact at 3.63 (CI 3.33–3.93) DALYs (Fig. 4c), mainly through low back pain (0.48 DALYs), ischemic heart disease (0.47), substance use disorder (0.37), COPD (0.23), depression (0.22) and neck pain (0.21).

**Extended Data Fig. 5:**
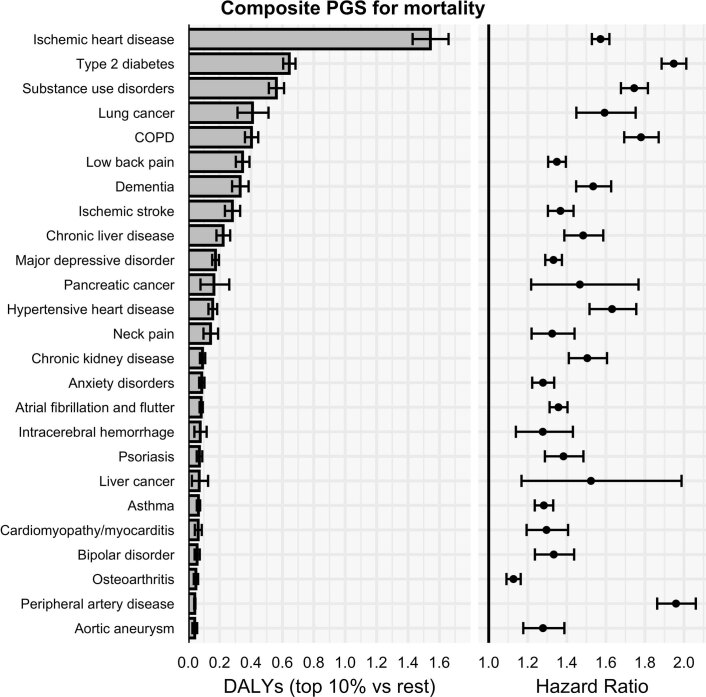
Attributable DALYs and HRs the composite PGS of mortality formed in FinnGen for top 25 diseases. The composite PGS for mortality was formed using all the 30 PGSes (Supplementary Table 10) to predict survival by emulating the approach by Meisner et al.^9^ Estimates are based on 309,136 individuals (FinnGen). Error bars denote 95% confidence intervals.

Following the methodology by Meisner et al.^9^ we used the 30 PGSs to predict mortality in a Cox model, then extracted the linear predictors for each individual from that model to form a composite PGS of mortality. Individuals in the top 10% versus bottom 90% of this composite PGS had a higher hazard of death at HR = 1.56 (CI 1.50–1.62) and the highest individual attributable DALYs at 6.52 (CI 6.15–6.88, Extended Data Fig. 5) out of all the PGSs.

### Sex-specific effects

**Fig. 5:**
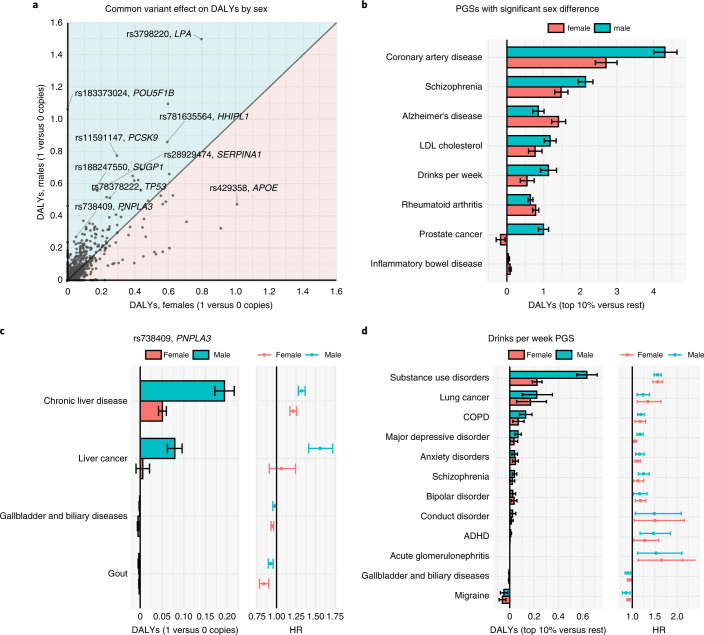
Sex-specific impact of common variants and polygenic scores on DALYs. **a**, Absolute attributable DALYs from carrying one versus zero copies of the minor allele separately for males (*n* = 331,211) and females (*n* = 404,537). **b**, PGSs (top 10% versus the rest) with a significant sex difference in attributable DALYs in FinnGen (males, *n* = 135,396; females, *n* = 173,740). **c**, For rs738409 (*PNPLA3*), we report the attributable DALYs and HRs for each disease by sex (males, *n* = 331,211; females, *n* = 404,537). **d**, Attributable DALYs and HRs by disease and sex for the PGS predicting drinks per week in FinnGen (males, *n* = 135,396; females, *n* = 173,740). Error bars denote 95% CIs.

We repeated some of the analyses stratified by sex (Supplementary Tables 5, 6 and 11–14). We observed significant sex differences in total DALYs at *P* < 0.05 for 474 (45%) of the common variants (Fig. 5a). Sex differences in attributable DALYs can result from differences in the effect of the genetic exposure on the disease or differences in DALYs attributed to men and women by the GBD^27^. rs738409 (PNPLA3-I148M), a missense variant in *PNPLA3* linked to liver fat accumulation and steatohepatitis^41^, provides a clarifying example: carrying one versus zero copies of the minor allele resulted in 0.27 (CI 0.24–0.30) attributable DALYs in males and 0.05 (CI 0.03–0.07) DALYs in females (sex difference *P* = 1.0 × 10^−34^). The sex difference is in part driven by differences in HRs (Fig. 5c) for chronic liver disease (HR = 1.32, CI 1.28–1.37 in males versus HR = 1.21, CI 1.17–1.26 in females) and, in part, because DALYs for chronic liver disease are higher in men than women^27^ (431 versus 158 yearly DALYs per 100,000; Supplementary Table 3).

Eight out of 30 PGSs exhibited significant sex differences in attributable DALYs (Fig. 5b). Most of the differences were explained by different DALYs attributed to men and women rather than differences in HRs. For example, the PGS for weekly alcohol consumption^42^ had similar HRs between sexes across all diseases (Fig. 5d) but markedly different effect on DALYs for substance use disorders reflecting the threefold higher population DALYs for men as estimated by the GBD^27^ (1,500 versus 497 yearly DALYs per 100,000; Supplementary Table 3).

### Population-attributable DALYs for common variants

**Fig. 6:**
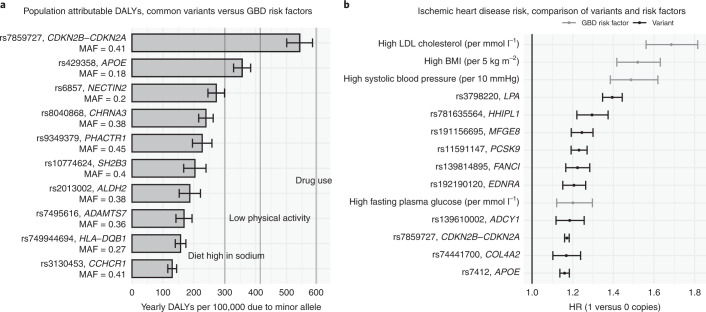
Effect of common variants on population-level DALYs in the Finnish population. **a**, Bars represent yearly population-level DALYs attributable to top-ten-ranking common variants. The vertical lines represent yearly population-level DALYs attributable to three selected modifiable risk factors as estimated by the GBD^11^ for Finland 2019. **b**, Top-ten HRs for ischemic heart disease of common variants and four HRs of modifiable risk factors as estimated by the GBD (50–54-year age group)^11^. Estimates are based on 735,748 individuals. Error bars denote 95% CIs.

Next, for the Finnish population, we estimated the amount of population-attributable DALYs per year per 100,000 from all (heterozygous and homozygous) carriers of the minor allele: the expected amount of healthy life years per year per 100,000 individuals in the population that would be gained if the minor allele were completely removed. rs7859727 (*CDKN2B-CDKN2A*) had the highest population-attributable DALYs, with minor allele carriers accounting for 447 (CI 420–473) yearly population DALYs per 100,000 in Finland 2019 (Fig. 6a). The large population effect of this variant is explained by its effect on ischemic heart disease (HR = 1.17, CI 1.16–1.18) and high frequency in the Finnish population (MAF = 0.41). Compared to population DALY estimates for modifiable risk factors from the GBD^11^ (Fig. 6a), the population-attributable DALY estimates of several common variants are similar to the total impact of a diet high in sodium (300 yearly population DALYs per 100,000), low physical activity (415) and drug use (595), but less impactful than the most important modifiable risk factors such as high systolic blood pressure (3,666), smoking (2,992) and high BMI (2,506)^11^. We also compared the HRs for ischemic heart disease between eight common variants and four modifiable risk factors (Fig. 6b). Clinically meaningful changes in modifiable risk factors (for example, 10 mm Hg higher systolic blood pressure) as estimated by the GBD, lead to comparable increases in ischemic heart disease risk (HR = 1.20 to 1.69)^11^ as having one versus zero copies of the risk variants (HR = 1.16 to 1.39).

### Additional population DALYs attributable to Finnish enrichment

**Extended Data Fig. 6:**
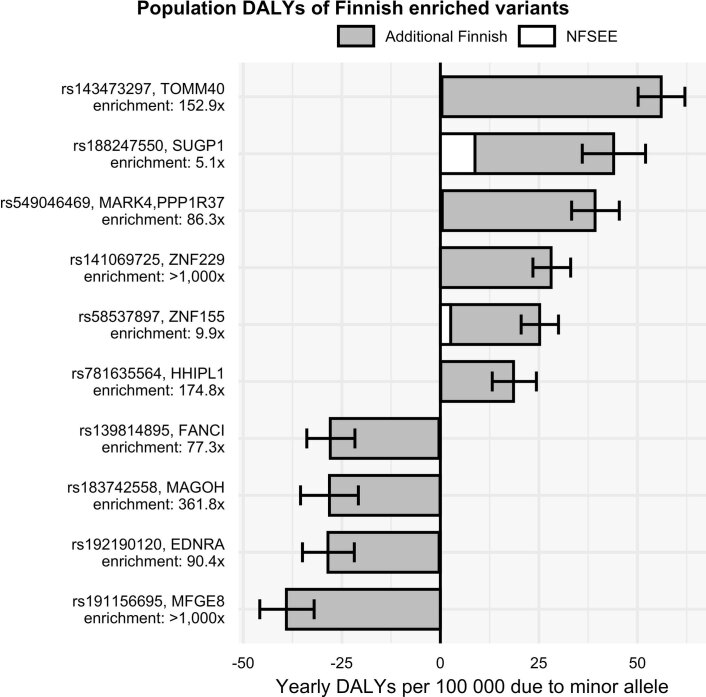
Impact of Finnish-enriched variants on population-level DALYs. The proportion of population DALYs accounted for by the most impactful 9 Finnish-enriched variants. The white part of the bars denotes the number of population DALYs attributable to the variant if the Finnish MAF were equal to the NFSEE MAF, the grey part represents additional population DALYs resulting from the enrichment in the Finnish population. Estimates are based on 309,136 individuals (FinnGen). Error bars denote 95% confidence intervals. NFSEE, non-Finnish-non-Swedish-non-Estonian Europeans.

Finland is a well-known example of an isolated population where multiple historical bottlenecks^43^ have contributed to the enrichment of several functional genetic variants^43,44^ otherwise rare in non-Finnish populations. We estimated the population-attributable DALYs that are due to the enrichment in the Finnish population compared to non-Finnish, non-Swedish, non-Estonian European (NFSEE) populations. The largest impact on population-attributable DALYs (Extended Data Fig. 6) was observed for rs143473297 (*TOMM40*) contributing to 56.1 (CI 50.1−62.0) yearly population DALYs per 100,000 individuals of Finnish ancestry through increased risk of dementia (0.57 individual-attributable DALYs, HR = 1.95; Supplementary Table 5). One remarkable example of a protective Finnish-enriched variant is rs191156695, an inframe insertion in *MFGE8* (ref*.* ^45^). The presence of the Finnish-enriched allele in the population contributes to preventing 39.1 (CI 32.0–45.8) yearly population DALYs per 100,000 individuals of Finnish ancestry (Extended Data Fig. 6) solely through decreasing ischemic heart disease risk (HR = 0.80, CI 0.77–0.84).

## Discussion

As genetic risk factors are becoming increasingly relevant to various fields of medicine, the ability to evaluate their impact on disease burden is crucial. In this study, by combining genetic information with DALYs from the GBD^27^, we present a comparative risk assessment approach^11^ for genetic risk factors, which makes it possible to uniformly compare the impact of genetic exposures through multiple diseases in terms of DALYs (‘lost healthy life years’). Overall, rare deleterious variants tended to have higher effects on DALYs than common variants at the individual level. Genetic exposures increasing the risk of ischemic heart disease tended to be most impactful in terms of DALYs (Supplementary Tables 5 and 6) as it accounts for the largest share of population DALYs in the GBD^27^ for Finland 2019 (11.5%; Supplementary Table 3).

The largest effects on individual DALYs were observed for deleterious rare variants in *BRCA1* (breast and ovarian cancer), *BRCA2* (breast, ovarian, liver and prostate cancer), *MYBPC3* (cardiomyopathy and myocarditis), *LDLR* (ischemic heart disease) and *MLH1* (colon and rectum cancer); however, due to the rarity of these variants, the population impact was, at most, 21 yearly population DALYs per 100,000 for *BRCA2* (Supplementary Table 8), which is substantially lower than for the top common variant rs7859727 (*CDKN2B-CDKN2A*) where minor allele carriers account for 447 yearly population DALYs per 100,000 (Supplementary Table 4).

Overall, the top PGSs exert their effect through cardiometabolic traits (for example, through ischemic heart disease for shorter lifespan^10^, coronary artery disease^46^ and type 2 diabetes^47^ PGSs) or pain/addiction-related traits (for example, through low back pain, substance use disorders, lung cancer and COPD for multisite chronic pain^40^, lower educational attainment^48^, major depressive disorder^49^ and smoking initiation^42^ PGSs; Supplementary Table 12). Note that the effect estimates for PGSs depend on the cutoff used. For the shorter lifespan PGS^10^, if we instead used top 1% versus the rest or top 50% versus the rest cutoffs, the individual DALYs would have been 5.60 and 2.76, respectively, instead of the reported 3.81 for top 10% versus the rest. Note also that the effects of PGSs depend on their predictive performance, so their relative importance can change and their effects on DALYs will increase as larger GWASs are used to construct them or methodological advances improve PGS performance.

Most common variants with the largest effects on DALYs affected ischemic heart disease risk (for example, rs3798220, *LPA*; rs11591147, *PCSK9*; and rs1537371, *CDKN2B-CDKN2A*) and some affected risk of dementia (rs429358, *APOE*), prostate cancer (rs183373024, *POU5F1B*) or type 2 diabetes (rs117361510, *JPH2*). The number of DALYs for a disease in the GBD^26^ is driven by how common it is, how much it contributes to premature mortality and how much it lowers quality of life. Common diseases that either lead to premature mortality (high YLLs, such as heart disease) and/or long periods of living with disability (high YLDs, such as low back pain) account for a large number of population DALYs (Supplementary Table 3). Consequently, genetic exposures increasing the risk of high-DALY diseases predominate the results. Some variants might affect DALYs by modifying intermediate risk factors such as BMI and blood pressure and, in our analyses, we have included several variants that were associated with six major modifiable risk factors. Nonetheless, the high-ranking variants are associated directly with the diseases rather than with intermediate risk factors (rs8040868 in *CHRNA5/A3/B4* being a notable exception due to its impact on smoking behavior).

**Extended Data Fig. 7:**
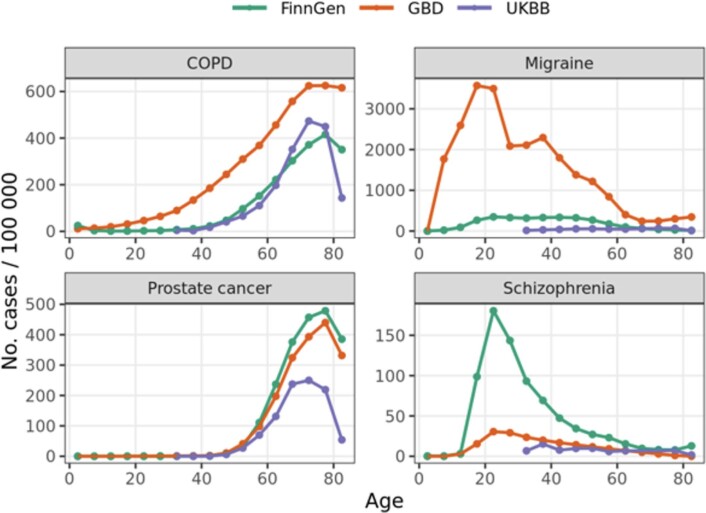
5-year incidence estimates of COPD, migraine, prostate cancer, and schizophrenia in FinnGen and UK Biobank (UKBB) compared to the population incidence estimates from the Global Burden of Disease Study 2019 for Finland 2019 (GBD). Number of new cases per 100,000 was calculated as number of new cases per 100,000 during each age interval divided by person-years contributed by individuals that were not cases in the earlier age intervals. The incidence of some diseases like prostate cancer is well captured in UKB and FinnGen, while other disease, such as migraine, as severely under-estimated. Schizophrenia incidence is elevated in FinnGen as one of the legacy cohorts recruited individuals with a psychotic illness.

There are multiple strengths and limitations to our study. One key strength is that the genetic associations were estimated using individual-level data from two large biobank studies with long registry-based follow-ups. We apply a shrinkage procedure to the associations and thus obtain a conservative overview of the pleiotropic effect of genetic exposures on 80 major diseases accounting for 83.1% of the total DALYs in Finland through all non-communicable diseases in 2019 (ref. ^27^). In quantifying the disease burden, we combine the association results with population DALYs from the GBD^27^, which reports perhaps the most accurate and unbiased estimates of population-level disease burden. This is important for many diseases for which defining the absolute amount of disease burden relying on available data is biased because of under-ascertainment (for example, migraine) or non-representativeness (for example, schizophrenia; Extended Data Fig. 7). One important aspect of the DALY estimates from the GBD is that the disease definitions are non-overlapping and the DALYs avoid double-counting by allowing a single cause of death when estimating the YLLs and through a comorbidity-correction procedure when estimating YLDs (Supplementary Appendix 1 Section 4.9 of GBD 2019 (ref. ^11^)). Finally, compared to the GBD risk factors approach^11^, which estimates attributable DALYs relying on effect estimates of the modifiable risk factors based mostly on (non-genetic) observational analyses, genetic exposures that we examine are less likely to be impacted by confounding and other biases, making a causal interpretation of our estimates more credible.

In Supplementary Table 15 we provide an overview of the study limitations, some of which can be overcome in future iterations of this work. Here we discuss perhaps the most important ones. First, we take it as given that the DALYs estimated by the GBD^27^ are accurate and that DALYs are a valid and meaningful measure of disease burden (which has been debated^50^). Second, the DALYs for individuals with a disease are assumed to be the same among those with and without the genetic exposures (for example, individuals with and without a damaging *BRCA1* mutation that develop breast cancer accumulate DALYs similarly). Third, we used DALYs estimated for Finland 2019 and estimated genetic associations using data between 1972 and 2020 in Finland and the United Kingdom, so the estimated effects on lifetime individual DALYs for someone born today can change as the disease incidence, medical care and mediating factors (for example, smoking) change. Fourth, although we suggest a causal interpretation for the attributable DALYs, there are important caveats. Despite rigorous statistical fine-mapping, the reported variants with the highest posterior probability might only tag underlying causal genetic variation. For example, the rs3798220 (*LPA*) variant is known to tag copy number variation of the Kringle IV-like domain 2 in the *LPA* locus, which is the probable mechanism behind the association of rs3798220 with ischemic heart disease^51^, so rs3798220 is not actually responsible for the effect on DALYs. Another caveat lies in the possibility that a reported variant does not tag one causal variant, but multiple causal variants in linkage disequilibrium (LD); however, using expression quantitative trait loci data, previous work on quantitative lipid traits has shown that a minority of pleiotropic effects at a given locus are explained by this configuration^52^. Fifth, note that we mainly present attributable DALYs for both sexes in aggregate, which might be misleading for exposures with sex-specific effects. For example, rs183373024 (*POU5F1B*) affected DALYs only through prostate cancer and benign prostatic hyperplasia (0.54 DALYs in aggregate, 1.06 in males and 0 in females; Supplementary Table 5). Finally, our genetic association results are estimated in participants of Finnish ancestry in FinnGen and European ancestry in UKB, which limits the generalizability to populations of non-European ancestry.

Although our results are presented using DALY estimates for Finland, limiting generalizability across countries, the presented framework can be applied to other countries and ancestries under certain assumptions. First, the effect sizes need to be similar in the target population. There is increasing evidence that many causal variants have similar effects across continental ancestry groups^53–57^, but this does not apply to PGSs^58^. Assuming effect sizes are consistent, a plausible assumption for populations of mostly European ancestry, one needs to know the frequency of the genetic exposures in the target population, which is particularly challenging in countries with a heterogeneous ancestry composition. In the absence of representative genetic surveys, it might be possible to use self-reported ancestry information combined with frequency datasets such as gnomAD^59^. With the effect estimates and frequencies, one can use the GBD estimates for the target country to derive localized estimates.

In summary, we present an approach to combine genetic association results with disease burden estimates from the GBD and provide an overview of the impact of genetic exposures on DALYs. We show that some common variants account for as many DALYs as some well-established modifiable risk factors and that PGSs are highly predictive of DALYs. For drug development, the attributable DALYs for a variant can provide an initial assessment of the potential impact of drugs targeting the relevant biological pathway, whereas extending the approach to the transcriptome-wide association study framework can provide information on potential drug effects in specific tissues. While genetic risk factors are not yet modifiable in practice, information on genetic risk can be used to target preventive measures and screening. Knowing the relative contribution of different genetic risk factors on disease burden can help prioritize and design interventions using genetic information. Approaches based on in vivo gene editing^19–21^ could use the information on expected DALYs prevented to prioritize potential targets for clinical trials and evaluate the effects of the intervention across the lifespan. Finally, the DALYs perspective can be useful for studies evaluating the utility of monogenic and polygenic embryo screening^22–26^ in assessing the potential impact and making tradeoffs between genetic risk for different diseases. To conclude, by translating information on genetic risk into expected healthy life years lost, genetic risk factors can be put in the larger context of traditional risk factors and compared in terms of their effect across multiple diseases, which enables the development and implementation of clinical applications utilizing genetic information in a more guided way.

## Methods

### General statistical methods

All *P* values are nominal. Error bars represent nominal 95% CIs. Confidence intervals for the disease-specific DALYs were generated using the delta method. Confidence intervals for total DALYs for each exposure were estimated by repeating 10,000 times the computation of total DALYs while resampling the log HR estimates from a multivariate normal distribution corresponding to the approximate sampling distribution of log HRs and by taking the 2.5th and 97.5th percentiles as the bounds for the 95% CIs (see below for detailed description). The sex-specific results were generated by estimating the HRs stratified by sex and using sex-specific population DALYs reported by the GBD^27^. For all error estimates we assumed that there was no uncertainty in frequencies of the exposures (for example, allele frequencies) or DALYs reported by the GBD, so we only considered variation from HR estimation in the error estimates.

### Cohort description

FinnGen is a public-private partnership research project combining genotype data generated from Finnish biobanks and digital health record data from Finnish health registries (https://www.finngen.fi/en). The Supplementary Information contains the full list of contributors. For a comprehensive description of the cohort and methods, see the FinnGen flagship manuscript^28^. Participants for FinnGen include participants of legacy cohorts, both population-based epidemiological cohorts (initiated as far back as 1992) and disease-based cohorts and volunteers from biobanks. All samples donated to Finnish biobanks are eligible for FinnGen. Information about the opportunity to donate a sample and medical information for biobank research was distributed by leaflets, advertisement campaigns in press and on TV and by dedicated biobank nurses in hospitals.

Like other Nordic countries, Finland has nationwide electronic health registers originally established primarily for administrative purposes to monitor healthcare usage^61^. These registers cover virtually all major health-related events, such as hospitalizations, prescription drug purchases, medical operations, cancers and deaths. All registers can be linked using the unique personal identity code which is given to every permanent resident of Finland. In this context, loss to follow-up can only result from emigration.

Registry data on all participants were collected from different national registers, including hospital and outpatient visits in HILMO, Care Register for Health Care (diagnoses, ICD-8–10; operations, NOMESCO Classification of Surgical Procedures), Causes of Death (immediate, contributing and underlying causes of death as ICD codes), reimbursed medication entitlements and all prescription medication purchases (ATC codes and special reimbursement codes) and the Finnish Cancer Registry (ICD-O-3 codes). The diagnostic accuracy and validity of these registers has been reviewed in many previous publications^62–66^.

We additionally included participants from the UKB, which is a population-based biobank cohort study that recruited around 500,000 people aged between 40 and 69 years from 2006 to 2010 across the United Kingdom. For details on participants and recruitment see Sudlow^29^ and Bycroft^67^. We used UKB data for common variants (meta-analysis with FinnGen) and rare variants (UKB only).

In FinnGen, we included participants from the Finnish ancestry passing genotypic quality control (data freeze 7) with *n* = 309,136 (56.2% female) with median (IQR) age at start of follow-up of 15.1 (0–26.3) and at end of follow-up 62.2 (47.1–72.9). The median (IQR) follow-up length was 47.3 (41.6–47.7) years (Supplementary Table 1). Follow-up for FinnGen started on 1 January 1972 and ended on 31 August 2020. For UKB we used participants of European ancestry passing genotyping quality control: *n* = 426,612 (54.1% female) with median (IQR) age at start of follow-up of 47.2 (39.5–52.4) and at end of follow-up 69.1 (61.7–74.2). The median (IQR) follow-up length was 22.3 (22.3–22.3) years (Supplementary Table 1). Follow-up for UKB started on 1 January 1998 and ended on 30 April 2020.

### DALYs

As the measure of disease burden, we used the DALYs from the 2019 GBD study^27^ with data publicly available at https://ghdx.healthdata.org/. DALYs are a metric for measuring population-level disease burden that combines a measure of premature mortality (YLL) and a measure of healthy life years lost due to lowered quality of life (YLD), so DALYs are the sum of YLDs and YLLs (Extended Data Fig. 1). The GBD is a longitudinal study that estimates the incidence, prevalence, mortality, YLLs, YLDs and DALYs due to various collectively exhaustive and mutually exclusive diseases and injuries (369 in 2019) for hundreds of countries (204 in 2019)^27^. GBD strives to model unbiased estimates using various sources of information, including census data, household surveys, civil registration and vital statistics, disease registries, health service use data and more. The estimation process for DALYs is complex and varies from disease to disease (see the 2019 GBD publication^27^ and its Supplementary Appendix 1 for a description of the methods).

Simplifying the GBD estimation process, the yearly YLDs in a population are estimated by multiplying the prevalence of a disease by its disability weight, which represents the magnitude of health loss due to living with the disease scaled between 0 (perfect health) and 1 (death). Yearly YLLs are estimated by multiplying the number of deaths attributable to a disease by the standard life expectancy at age of death^27^. The DALYs estimated by GBD avoid double-counting (for YLLs, there can only be one cause of death and for YLDs they implement a comorbidity correction^27^). Because all of the genetic exposures, except rare variants, were measured in FinnGen, we used the 2019 GBD metrics for Finland and present all estimates for Finland (see Supplementary Table 3 for a list of DALYs, YLLs and YLDs for the included diseases).

### Disease phenotypes

To define similar disease phenotypes in FinnGen and UKB as those in the GBD study, we manually mapped 89 non-overlapping non-communicable diseases from the 2019 GBD study^27^ into FinnGen clinical end points (Supplementary Table 2). For more information on the FinnGen clinical end points, please see the FinnGen flagship manuscript^28^. The clinical end points can be explored in https://risteys.finngen.fi/. The mapping was performed by matching the disease categories in the GBD as closely as possible to existing single FinnGen clinical end points or combinations of multiple FinnGen end points (separated with ‘|’ operator in Supplementary Table 2 FinnGen end points column).

We did not attempt to map infectious diseases, accidents, injuries, sufficiently rare conditions, conditions not ascertainable through registries or conditions for which the GBD 2019 does not report DALYs for Finland. Due to statistical power considerations, we removed 9 conditions for which there are under 500 cases, ending up at 80 medical conditions, which account for 83.1% of yearly total population-level DALYs from non-communicable diseases in Finland 2019 (Supplementary Table 3).

For example, the ischemic heart disease definition in GBD through causes of death encompasses ICD-10 codes I20–I25.9, but our definition of ischemic heart disease in FinnGen consists of combining five FinnGen end points: I9_MI (myocardial infarction, IC10-codes I21–I22, ICD-9 and ICD-8 code 410), I9_MI_COMPLICATIONS (complications following myocardial infarction, ICD-10 code I23), I9_POSTAMI (status post-acute myocardial infarction, ICD-10 code I25.3, ICD-9 and ICD-8 code 412), I9_CORATHER (coronary atherosclerosis, ICD-10 codes I24-I25, T82.2 and Z95.1) and I9_REVASC (coronary revascularization, defined through NOMESCO operation codes for coronary angioplasty and coronary artery bypass grafting). The first occurrence of any of these five end points was coded as the event of ischemic heart disease.

For FinnGen, we did not define disease phenotypes just using the ICD-10 codes provided in GBD for multiple reasons. First, ICD-10-based definitions for all disease conditions in GBD are not available (for example, low back pain). Second, even when GBD presents ICD-10 diagnoses, as follow-up for FinnGen for this study started in 1968, diseases need to also be defined through ICD-8 (in use until Dec 1987) and ICD-9 (in use from Jan 1987 to Dec 1995). Third, the FinnGen clinical end point phenotyping algorithms have been hand-crafted to utilize various sources of registry data in addition to diagnosis codes (for example, operation codes, prescription drug purchases and special drug reimbursement codes for certain diseases).

For example, migraine is a disease commonly managed in the primary care setting, so relying on diagnosis codes present in hospital and secondary care specialist visits data will lead to serious under-ascertainment of migraine. Also, there is misclassification of other types of headaches as migraine. The FinnGen end point that we used to define migraine (MIGRAINE_TRIPTAN) requires that a patient has at least one purchase of prescribed triptans, capturing, for example, patients who do not have a diagnosis code for migraine in the available registries that do not include primary care.

Phenotyping in FinnGen^28^ relied on information on diagnoses starting from 1972 in the hospital discharge registry^65^, the causes of death registry and the cancer registry. The drug purchases registry was additionally used starting from 1995 for selected diseases (for example, migraine). The cumulative incidence of the diseases varied from 14.93% (cataract) to 0.16% (acute glomerulonephritis) in FinnGen. Phenotyping in UKB was performed via groups of ICD-10 codes mapped from the FinnGen end points, relying on ICD-10 diagnosis codes from the hospital episode statistics, cancer registry and causes of death registry data starting from 1998 (Supplementary Table 2). Due to statistical power considerations, we analyzed the following diseases only in FinnGen as in UKB there were fewer than 500 cases: hemoglobinopathies and hemolytic anemias (*n* = 480 in UKB), Hodgkin lymphoma (*n* = 464), autism spectrum disorders (*n* = 164), eating disorders (*n* = 163), acne vulgaris (*n* = 106), acute glomerulonephritis (*n* = 58), ADHD (*n* = 26) and conduct disorder (*n* = 13). As both FinnGen and UKB rely mainly on diagnosis codes recorded at hospitals, conditions that are usually managed in the primary or outpatient care setting are under-ascertained, but this does not bias the attributable DALY estimates if the HR estimates are unbiased.

### Genotyping, imputation and quality control

Samples for FinnGen were genotyped using Illumina and Affymetrix arrays (Illumina and Thermo Fisher Scientific). Genotype calls were made with GenCall or zCall for Illumina and the AxiomGT1 algorithm for Affymetrix data. Chip genotyping data produced with previous chip platforms and reference genome builds were lifted over to build v.38 (GRCh38/hg38) following the protocol described in ref. ^68^. Participants with ambiguous sex, genotype missingness of over 5%, excess heterozygosity (±4 s.d.) and non-Finnish ancestry were excluded. Variants with over 2% missingness, low Hardy–Weinberg equilibrium *P* value (<10^−6^) and minor allele count <3 were excluded. Array data pre-phasing was performed with Eagle v.2.3.527 with default parameters, except the number of conditioning haplotypes was set at 20,000. Genotype imputation was performed with Beagle v.4.128 as described in ref. ^69^ by using the SISu v.3 reference panel developed from data on 3,775 high-coverage (25–30×) whole-genome sequenced Finns. For more details, please see the FinnGen flagship manuscript^28^.

For detailed information on genotyping, imputation and quality control for the UKB data, see Bycroft^67^. The genotyping was performed using the Applied Biosystems UK BiLEVE Axiom Array or the Applied Biosystems UKB Axiom Array. The genotype imputation was performed using a combination of the Haplotype Reference Consortium, UK10K and 1000 Genomes Project phase 3 reference panels by IMPUTE4 software. Variants with INFO score ≤ 0.8, MAF ≤ 0.01 and Hardy–Weinberg equilibrium *P* value ≤ 1 × 10^−10^ were excluded.

For variant annotation, we utilized the Variant Effect Predictor^60^ (VEP) v.103. For coding variants, we chose a single most severe consequence and corresponding gene among canonical transcripts. We considered stop_gained, frameshift_variant, splice_donor, splice_acceptor, missense_variant, start_lost, stop_lost, inframe_insertion and inframe_deletion as protein-truncating variants.

### Principal components and genetic ancestry assignment

For FinnGen, the principal component analysis (PCA) for population structure was performed using the following approach. First, the following filters were applied: (1) exclusion of chromosome 23; (2) exclusion of variants with INFO score 0.95; (3) exclusion of variants with missingness >0.01; (4) exclusion of variants with MAF < 0.05; and (5) LD pruning with window 500 kb, step 50 kb and *r*^2^ filter of 0.1.

The imputed genotypes were merged with 1000 Genomes Project phase 3 data into a single dataset of 49,451 pruned single-nucleotide polymorphisms (SNPs), on which the principal components (PCs) were estimated. An unsupervised Bayesian algorithm (Aberrant) was used to spot outliers in the PCA space and remove them. While this method automatically detected the 1000 Genomes Project samples with non-European and southern European ancestries as outliers, it did not manage to exclude some samples with western European origins. As the signal from these samples would have been too small to allow a second round to be performed without detecting substructures of the Finnish population, another approach was used. The FinnGen samples that survived the first round were used to compute another PCA. The European and Finnish 1000 Genomes Project samples were projected onto the space generated by the first three PCs. For each sample, the probability of belonging to the EUR/FIN cluster was estimated through using a chi-squared distribution based on the Mahalanobis distance to the centroid of each cluster. Samples whose relative probability of being part of the Finnish cluster was >95% were classified as belonging to the Finnish ancestry and retained in all following analyses for FinnGen (*n* = 309,136).

PCA and ancestry assignment in UKB followed the procedure of the Pan-UKB analysis (https://pan.ukbb.broadinstitute.org/) and the procedure is described at https://pan.ukbb.broadinstitute.org/docs/qc#ancestry-definitions.

### HLA imputation in FinnGen

The HLA imputation is described in detail elsewhere^70^. Briefly, HLA typing on 1,150 Finnish samples was performed by the HLA Laboratory of the Finnish Red Cross Blood Service using procedures accredited by the European Federation for Immunogenetics. Allele assignment of the seven HLA genes at two-field resolution level (unique protein sequence level) was performed by polymerase chain reaction (PCR)-based methods. HIBAG^71^ v.1.14.0 with 100 classifiers for each of the seven HLA genes was fitted using the training data of 1,150 individuals to construct an imputation reference for the Finnish population, which was used to impute the HLA alleles in FinnGen^70^. We analyzed 74 alleles in seven HLA genes (Supplementary Table 14) for the HLA region (chr6:29691116 to chr6:3054976 in GRCh38).

### Statistical fine-mapping

Summary statistics for fine-mapping were obtained from standard FinnGen pipeline summary statistics, where mixed-model logistic regression using SAIGE^72^ was used to obtain summary statistics for each FinnGen end point used to define the 80 diseases (Supplementary Table 2). The models used sex and age as precision covariates. Genotyping batch and the ten first genetic PCs were used to control for confounding due to population stratification and batch effects. Using the summary statistics, we fine-mapped all regions with at least one variant having *P* < 10^−8^ and extended the regions 1.5 Mb upstream and downstream from each lead variant. Overlapping regions were merged and used in SuSiE^30^ fine-mapping, allowing for up to ten causal variants per region and constructing 95% credible sets for each independent signal. In-sample dosage LD was computed using LDStore2. The FinnGen fine-mapping pipeline is available at https://github.com/FINNGEN/finemapping-pipeline.

The fine-mapping in UKB using SuSiE followed a similar procedure. Regions for fine-mapping were defined by greedily starting with the most significantly associated (highest chi-squared) variant, including all genome-wide significant (*P* < 10^−8^) variants within a window of 3 Mb centered at the variant and merging overlapping regions. Summary statistics were obtained using BOLT-LMM^73^ and SAIGE^72^. In-sample dosage LD was estimated using LDStore2. The maximum number of causal variants for each locus was ten. We only considered fine-mapping results for six quantitative risk factor traits (BMI, HbA1c, HDL cholesterol, LDL cholesterol, systolic blood pressure and cigarettes per day) and considered variants with *P* < 10^−12^ to restrict the number of variants to be selected.

### Common variants

We selected an initial list of 2,562 common variants (MAF > 0.01) for inclusion in the analysis that either (1) had at least one *P* < 5 × 10^−8^ association with any of the 80 diseases and had the highest probability of being causal within a SuSiE fine-mapped^30^ 95% credible set in FinnGen; (2) were coding variants with a *P* < 5 × 10^−8^ association with at least one of the diseases in FinnGen; or (3) had at least one *P* < 5 × 10^−12^ association with any of the selected six modifiable risk factor traits (BMI, HbA1c, HDL cholesterol, LDL cholesterol, systolic blood pressure and cigarettes per day) and had the highest probability of being causal within a SuSiE fine-mapped 95% credible set in UKB^31^. We labeled common variants as Finnish-enriched if they had at least fivefold MAF enrichment in FinnGen compared to NFSEE ancestry MAF in gnomAD^59^ and the NFSEE MAF was <0.01.

The 2,562 common variants in the initial list for inclusion can be in high LD with each other because we use fine-mapping results and coding variants for multiple phenotypes to select them. To select a set of common variants that are independent of each other for analysis, we performed LD clumping of the 2,562 variants as follows: We used PLINK^74^ v.1.90 to clump all common variants using an *r*^2^ threshold of 0.2, a 250-kb clumping window and FinnGen release 4 genotypes as the reference panel to remove SNPs in LD with variants having a smaller minimum *P* value among the 80 HRs for all examined diseases (meta-analysis estimates combining FinnGen and UKB); however, if there was a coding variant among the variants in LD, that was instead kept and others were discarded. Additionally, if there was a Finnish-enriched variant among the variants in LD (but no coding variant), the Finnish-enriched variant was kept. This resulted in 1,044 independent common variants to be included in the analyses (Supplementary Table 4). For each variant, we defined the minor allele to be the allele less common in FinnGen. Due to symmetry in attributable DALY estimation, going from one to zero copies versus zero to one copies of an allele only changes the sign of the individual-attributable DALYs estimates. Additionally, we determined six haplotypes (Supplementary Table 13) for *APOE* based on rs429358 and rs7412 alleles^34^.

### Rare deleterious variants

To analyze the effects of rare deleterious variants on DALYs, we used whole-exome sequencing data from a subset of participants in UKB (*n* = 174,379) from the December 2020 release. Data was preprocessed using hail v.0.2. We used the quality-controlled PLINK^74^ files provided by UKB. Variants were annotated using the Ensembl Variant Effect Predictor (VEP)^60^ following the approach in gnomAD^59^ (gnomadvep.vep_or_lookup_vep). VEP annotations were processed using the function ‘gnomadvep.process_consequences’, consistently with the gnomAD definition of pLOF, missense and synonymous variants, using the canonical transcript. We also extracted ClinVar-annotated^37^ variants (accessed in November 2020) and germline variants in *BRCA1* and *BRCA2* (accessed in November 2020) annotated by the ENIGMA consortium^38^. From ClinVar we considered ‘pathogenic’ and ‘likely-pathogenic’ variants (no filtering on star status or number of submitters) and from ENIGMA we considered ‘pathogenic’ variants. Variants with a frequency >0.001 were excluded because they are less likely to be deleterious. We considered all genes part of the ACMG recommendations for reporting incidental findings in clinical exome and genome sequencing studies^36^. We formed two types of rare variant burdens for individuals for each gene: (1) the pLOF burden was set as positive if there was at least one variant annotated as pLOF; and (2) the ClinVar/ENIGMA burden was set as positive if there was at least one variant annotated as ‘pathogenic’ in ENIGMA^38^ for *BRCA1* and *BRCA2* and for other genes ‘likely pathogenic’ or ‘pathogenic’ in ClinVar. Due to statistical power considerations, we restricted our analysis so that at least 35 individuals had a positive burden, resulting in nine genes for both burden types (Supplementary Table 7).

### Polygenic scores

We included 30 genome-wide PGSs for traits of interest constructed from publicly available summary statistics (Supplementary Table 10). We selected PGSs for psychobehavioral traits (for example cognitive ability and neuroticism), major chronic diseases (for example coronary artery disease and depression) and major risk factors (for example LDL cholesterol and blood pressure) to cover traits of interest with high-quality summary statistics available. PGSs were only analyzed in FinnGen, as many of the original summary statistics included UKB.

We used PRS-CS^75^ for generating the PGSs using external LD reference panel (1000 Genomes Project Europeans). We used the PRS-CS-auto algorithm, which learns the global scaling parameter ϕ from the data and performs well with large datasets. Default PRS-CS parameters were used and only HapMap 3 variants were considered (https://github.com/FINNGEN/CS-PRS-pipeline). Scores for lifespan, educational attainment, cognitive performance and intelligence were reversed before analysis to make all scores on net deleterious in terms of DALYs. For defining the exposure for survival models, we coded individuals for each PGS as one if they were in the top 10% of the score distribution and zero if not. Consequently, the individual DALYs can be interpreted as the effect on lifetime DALYs if the average individual in top 10% of the PGS were to have their PGS be that of the average in the bottom 90% of the PGS.

We additionally formed a composite PGS of mortality using all the 30 PGSs (Supplementary Table 10) emulating the approach by Meisner et al.^9^ by modeling survival via a Cox proportional hazards model with sex, the 30 PGSs and ten PCs of population structure as predictors. We then extracted the linear predictor for all individuals, while setting sex to 0.5 and the PCs to 0 (the mean) and interpreted these values as a composite PGS of mortality.

### Survival models

To estimate the HRs between all genetic exposure–disease pairs we used Cox proportional hazards regression via the coxph function in the survival package v.3.2-11 in R. The model was additive for allele counts. Sex and the first ten PCs of population structure were included as covariates. We used calendar age as the timescale and age at first record of the disease in the registries as time-to-event. Individuals were censored at death, emigration or end of registry-based follow-up (31 August 2020 in FinnGen and 30 April 2020 in UKB). For the common variants, HRs were estimated both in FinnGen and UKB separately and combined using fixed effects inverse-variance-weighted meta-analysis. A comparison of effect sizes between FinnGen and UKB is provided in Extended Data Fig. 3. We did not account for left censoring or relatedness of the individuals due to computational limitations. For the rare variant burden analysis in UKB, we used Cox regression with Firth’s Penalized Likelihood^76^ via the coxphf package v.1.13.1 and included sex and the four first genetic PCs as covariates.

As a sensitivity analysis in FinnGen, we examined whether accounting for relatedness would meaningfully change the standard errors of the log HRs. For 2,562 common variant–disease pairs we estimated the log HRs using a survival model clustered by family indicator to generate robust standard errors. We generated a family indicator from genotype data using KING^77^ v.2.2 only including HapMap 3 variants. Individuals up to a third degree of relatedness were included in the same family. The robust standard errors were estimated using a family indicator to compute robust standard errors by including cluster(family_id) as a covariate in coxph. Compared to the main analysis estimates, robust standard errors were a median 1.0128-times (IQR 1.0044–1.0205) larger. Thus, accounting for relatedness would not meaningfully affect the CIs and *P* values.

**Extended Data Fig. 8:**
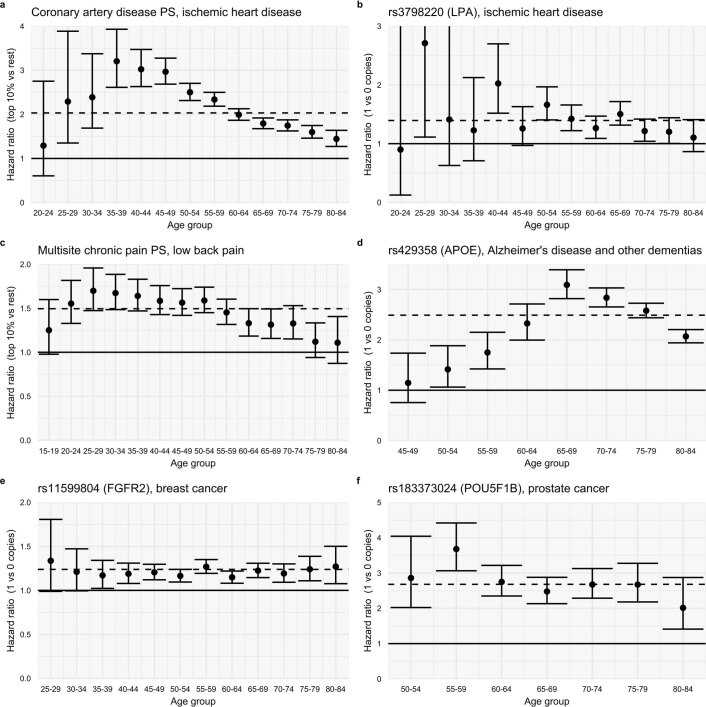
Hazard ratios by age group in FinnGen. For each age group, we estimated the HRs via a Cox proportional hazards model including individuals whose follow-up started before the beginning of the age interval that did not have a previous record of the disease. Dashed horizontal lines indicate the overall HR in FinnGen. The figure demonstrates age-varying HRs, especially for ischemic heart disease, Alzheimer’s disease and other dementias, and low back pain (**a,b,c,d**). For breast cancer and prostate cancer, the HRs were approximately constant across age groups (**e,f**). Estimates are based on 309,136 individuals (FinnGen). Error bars denote 95% confidence intervals.

As a second sensitivity analysis, we explored whether the effect of the genetic exposures on the diseases was age-dependent by performing age-stratified survival analyses for a subset of genetic exposures. Perhaps unsurprisingly^78^, we observed age-varying HRs for genetic exposures (Extended Data Fig. 8). For example, for the coronary artery disease PGS^46^, the HRs were 2.50 (CI 2.31–2.70) for the 50–54-year age group and 1.75 (CI 1.62–1.87) for the 70–74-year age group.

### Shrinkage

We use prior information to apply a shrinkage procedure to the HRs for exposure–disease pairs to reduce the effect of sampling variation at the cost of being more conservative (biasing total attributable DALYs toward zero). The possible benefits of shrinkage include: (1) the top-ranking variants suffer less from the Winner’s curse (overestimation due to sampling variation); (2) by reducing the number of diseases through which a variant contributes to DALYs, variants increasing risk of low-DALY diseases do not have their total DALY estimates overshadowed by noisy weak effects through high-DALY diseases reflecting sampling variation; and (3) the shrinkage helps remove possible weak effects due to residual confounding from population stratification.

We denoted by *b*_*e*,*d*_ the log HR of genetic exposure *e* on disease *d* = *1,..,80*. One genetic exposure at a time, we set the prior distribution of *b*_*e*,*d*_, to be a mixture distribution between the point mass at 0 and a 50:50 mixture of two normal distributions with means at −0.3 and 0.3, respectively and with a s.d. of 0.1. We denote the mixture weight of the non-zero component by *p*_*e*_, which can be interpreted as the exposure-specific proportion of non-zero effects across the 80 diseases. We set the prior distribution of *p*_*e*_ to a ${{{\mathrm{Beta}}}}\left( {\alpha = 1,\beta = 19} \right)$ distribution that has an expected value of 0.05. The full probability model is:

$$\begin{array}{l}p_e\sim {{{\mathrm{Beta}}}}\left( {\alpha ,\beta } \right),\pi _{e,d}\sim {{{\mathrm{Bernoulli}}}}\left( {0.5} \right),\\ b_{e,d}\sim {{{\mathrm{Bernoulli}}}}\left( {p_e} \right)\left( {\left( {1 - \pi _{e,d}} \right){{{\mathcal{N}}}}\left( {\mu ,\sigma ^2} \right) + \pi _{e,d}{{{\mathcal{N}}}}\left( { - \mu ,\sigma ^2} \right)} \right),\end{array}$$

where *α* = 1, *β* = 19, *μ* = 0.3 and *σ* = 0.1.

**Extended Data Fig. 9:**
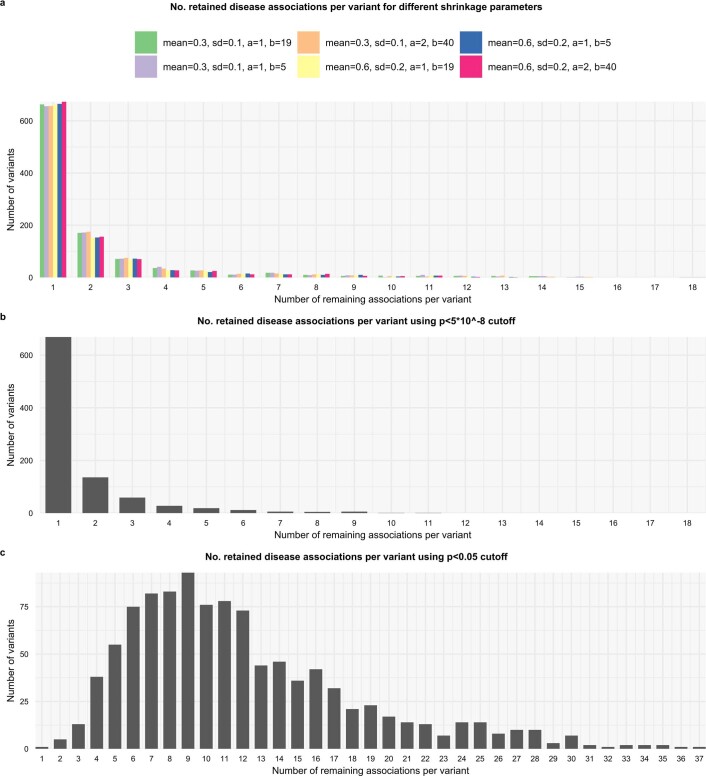
Histograms of the number of retained disease associations for common variants. Panel **a**: after shrinkage using different prior parameters, ‘mean’ and ‘sd’ correspond to the *μ* and *σ* prior parameters, ‘a’ and ‘b’ correspond to the *α* and *β* prior parameters. Panels **b** and **c**: using *P* < 5×10^−8^ or *P* < 0.05 cutoff instead of shrinkage. Overall, the distribution of retained association is not sensitive to the choice of prior parameters and is rather similar compared to using a *P* < 5×10^−8^ cutoff instead of shrinkage.

This model implies that, before seeing the data, for each genetic exposure, we expect a non-zero effect for four (= 0.05 × 80) diseases and the direction of the non-zero effects are equally likely to be risk increasing (centered around HR = 1.35) as protective (centered around HR = 0.74). In practice, which effects are shrunk to zero and which are retained as non-zero, does not vary considerably when these prior parameters are varied (Extended Data Fig. 9). For each genetic exposure *e* at a time, we used a Markov Chain Monte Carlo procedure with 10,000 iterations to estimate the posterior probabilities of the log HRs (*b*_*e,d*_) for diseases *d* = 1,2,…,80 coming from the null model (point mass at zero). We discarded any log HRs where the null probability was over 10% and, for the remaining log HRs, we used the maximum partial likelihood estimates from the Cox proportional hazards model in the downstream analyses.

### Examining shrinkage performance using simulated data

As a sensitivity analysis to explore the performance of the shrinkage method, we used hail v.0.2 to simulate variant–phenotype associations for 80 diseases where the true underlying effects are known. The approach used genetic data from 361,194 individuals from UKB with European ancestry and has the advantage of using realistic variant frequencies and population structure as compared to simulated genetic data. Only 558,240 independent HapMap 3 SNPs were considered in the analysis. Using the ldscsim.simulate_phenotypes function we simulated 80 phenotypes based on a spike-and-slab model with a different probability of SNPs being causal (*π*). The heritability of the phenotypes was randomly sampled from a uniform distribution ranging from 10 to 60%. The phenotypes were consequently binarized based on the disease prevalence observed in FinGen using the function ldscsim.binarize. We considered four *π* values (0.001, 0.002, 0.005 and 0.01) meaning that 0.1%, 0.2%, 0.5% and 1% of the 558,240 independent variants had a true underlying effect different from 0.

We then ran a GWAS for each of the phenotypes across the four different *π* scenarios. This allows us to obtain an observed effect size from the GWAS and an expected true underlying effect. Out of the observed effects, consistently with the variant selection process used on the real data, we only included variants that had at least one genome-wide significant association (*P* < 5 × 10^−8^). For this selected group of variants, we applied the same shrinkage procedure as in the main analysis.

**Extended Data Fig. 10:**
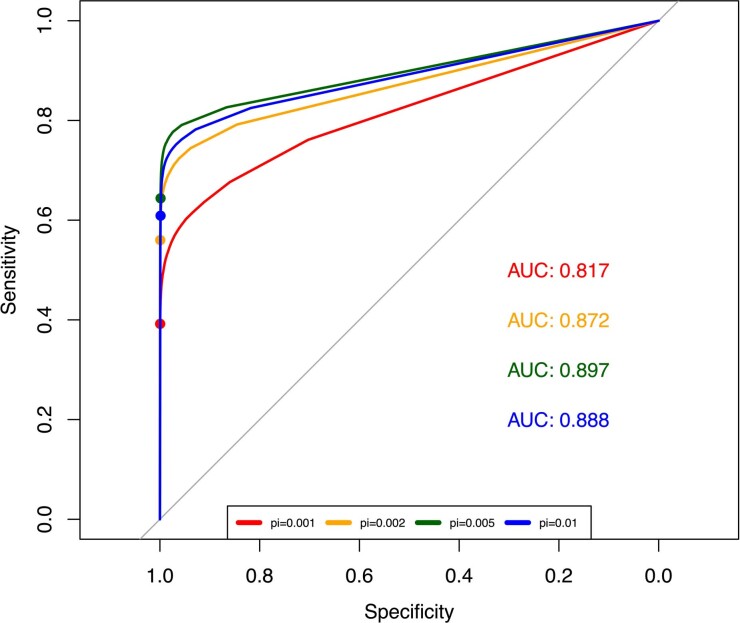
Receiver operating characteristic curves using our shrinkage method as a classifier to identify true causal variants in simulated data. Using *hail*, we simulated GWAS summary statistics for 80 binary phenotypes with heritability sampled uniformly between 0.1 and 0.6. We repeated this for four scenarios where the probability of a variant being causal (pi) is 0.001, 0.002, 0.005, and 0.01. We then applied our shrinkage procedure and classified those variants with a posterior probability of being from the null model of <10% as being causal (colored dots on lines represent this threshold). See Supplementary Table 16 for details on classification performance.

Our procedure shrinks most of the variant–phenotype associations to 0, while maintaining others unshrunk. Because we know the true underlying effect sizes, that is, which variants have effect size of 0 (null model) and effect sizes different from 0 (alternative model), we can compare how well our procedure shrinks variants from the null model versus does not shrink those from the alternative model. Overall, our approach results in area under the curve values of 0.817 to 0.897 for different values of *π*. Thus, the shrinkage approach can identify true causal variants in simulated GWAS data (Extended Data Fig. 10 and Supplementary Table 16) reasonably well.

### Attributable DALYs

Similarly to the GBD^11^, we used the HRs and frequencies of the exposures to estimate attributable DALYs one disease at a time. We used the multilevel exposure formula^79^ for the population-attributable fraction (the fraction of cases of disease *d* caused by the exposure levels in the population deviating from counterfactual levels):

$${{{\mathrm{Population}}}}{\mbox{-}}{{{\mathrm{attributable}}}}{\mbox{-}}{{{\mathrm{fraction}}}} = {{{\mathrm{AFp}}}}_d = \frac{{\mathop {\sum}\nolimits_i {{{{\mathrm{P}}}}_i{{{\mathrm{HR}}}}_{d,i}} - \mathop {\sum}\nolimits_i {{{{\mathrm{P}}}}_i^\prime {{{\mathrm{HR}}}}_{d,i}} }}{{\mathop {\sum}\nolimits_i {{{{\mathrm{P}}}}_i{{{\mathrm{HR}}}}_{d,i}} }}$$

where ${{{\mathrm{HR}}}}_{d,i}$ is the HR for disease *d* at exposure level *i* (for example, one copy) relative to reference (for example, zero copies) and _*Pi*_ is the fraction of the population at exposure level *i* and ${{{\mathrm{P}}}}_i^\prime$ represents the fraction of the population at exposure level *i* in the counterfactual scenario (for example, if all with one copy had zero instead: ${{{\mathrm{P}}}}_1^\prime = 0$, ${{{\mathrm{P}}}}_0^\prime = {{{\mathrm{P}}}}_0 + {{{\mathrm{P}}}}_1$ and ${{{\mathrm{P}}}}_2^\prime = {{{\mathrm{P}}}}_2$).

As an example, for individuals carrying zero, one and two alleles with respective population frequencies of *P*_0_ = 0.7, *P*_1_ = 0.2 and *P*_2_ = 0.1 and HRs for disease *d* of ${{{\mathrm{HR}}}}_{d,0} = 1.00$, ${{{\mathrm{HR}}}}_{d,1} = 1.35$ and ${{{\mathrm{HR}}}}_{d,2} = 1.82$. For calculating attributable DALYs from individuals carrying one versus zero copies of the allele, define the counterfactual frequencies as ${{{\mathrm{P}}}}_0^\prime = 0.9$, ${{{\mathrm{P}}}}_1^\prime = 0.0$ and ${{{\mathrm{P}}}}_2^\prime = 0.1$ (making all with one copy have zero copies instead). Plugging these numbers into the AFp_*d*_ formula produces the population-attributable fraction of disease cases from those carrying one versus zero copies of the allele (the fraction of cases that would be prevented if all with one allele had zero instead), which is 0.061 in this case, so approximately 6.1% of disease cases would be prevented if all with one copy had instead zero copies at birth.

We then assumed that the estimated population-attributable fraction of disease cases can be interpreted as the population-attributable fraction of DALYs, which is true if all disease cases contribute on average the same amount of DALYs independent of whether they have the genetic exposure or not. This assumption does not hold if, for example, deleterious *BRCA1* mutation carriers develop breast cancer earlier and consequently accumulate more DALYs per case. Then, multiplying the population-attributable fraction of DALYs (AFp_*d*_) by the population DALYs per year per 100,000 reported by the GBD (DALY_*d*_ gives the population-attributable DALYs), interpreted in our example as the expected loss of healthy life years per year per 100,000 if the population frequencies of the exposure were ${{{\mathrm{P}}}}_i^\prime$ instead of _*Pi*_ (in our example, all with one copy had zero copies instead).

$${{{\mathrm{Population}}}}\,{{{\mathrm{attributable}}}}\,{{{\mathrm{DALYs}}}}_{{{\mathrm{d}}}} = {{{\mathrm{AFp}}}}_{{{\mathrm{d}}}} \times {{{\mathrm{DALY}}}}_d$$

We further estimated individual-attributable DALYs for binary counterfactuals (for example, having one versus zero copies, being in the top 10% of a PGS versus the bottom 90%) by dividing the population-attributable DALYs per 100,000 by the number of individuals with the exposure out of 100,000 (100,000 × _*Pi*_) and multiplying by life expectancy at birth (L):

$$\begin{array}{*{20}{l}} {{{{\mathrm{Individual}}}}\,{{{\mathrm{attributable}}}}\,{{{\mathrm{DALYs}}}}_{{{\mathrm{d}}}}} \hfill & = \hfill & {\frac{{{{{\mathrm{AFp}}}}_{{{\mathrm{d}}}} \times {{{\mathrm{DALY}}}}_d}}{{100,000 \times {{{\mathrm{P}}}}_i}} \times }{{{\mathrm{L}}}} \hfill \\ \hfill & {} \hfill {{{\mathrm{=}}}} & {\frac{{{{{\mathrm{Population}}}}\,{{{\mathrm{attributable}}}}\,{{{\mathrm{DALYs}}}}_{{{\mathrm{d}}}}}}{{{{{\mathrm{No}}}}{{{\mathrm{.individuals}}}}\,{{{\mathrm{exposed}}}}\,{{{\mathrm{per}}}}\,{{{\mathrm{100,000}}}}}} \times {{{\mathrm{L}}}}} \hfill \end{array}$$

where DALY_*d*_ represents the population DALYs per year per 100,000 through disease *d* from GBD, _*Pi*_ is the fraction of population for which the exposure is changed (for example, fraction of those with one copy) and L is included to convert yearly DALYs into lifetime estimates. These individual DALYs are interpreted as the expected loss of healthy life years for an individual caused by having the genetic exposure at birth. Finally, both population-attributable DALYs and individual DALYs can be summed up across the 80 diseases to arrive at the total impact of the genetic exposure. Note that the attributable DALYs (or the population-attributable fractions) for multiple exposures cannot be added together to estimate the effect of jointly intervening on multiple exposures^80^. Thus, summing the attributable DALYs for multiple genetic exposures does not prove a correct estimate for the counterfactual joint intervention on multiple exposures (for example, summing attributable DALYs of two variants). Also see Witte et al.^81^ for discussion on how population-attributable fractions relate to other measures of genetic contribution.

### Uncertainty estimation of total attributable DALYs

Assuming that there is no uncertainty in the DALY estimates from GBD and the estimated population prevalence of the exposures (for example, allele frequencies), for a single disease both attributable individual and population DALYs are a deterministic function of the HRs between the exposure and the diseases. Therefore, CIs for the effect of a genetic exposure on attributable DALYs through one disease was estimated using the delta method.

Estimating the total attributable DALYs through the 80 examined diseases is less straightforward, as the HRs for different diseases are not independent (for example, ischemic heart disease and lower extremity peripheral artery disease are comorbid, so risk variants tend to increase risk for both). Bootstrapping was not computationally feasible, so we estimated the uncertainty via resampling the multivariate normal distribution of the log HR estimates.

Considering a single genetic exposure *e*, let ${{{\boldsymbol{b}}}}_e = (b_{e,1}, \ldots ,b_{e,80})^{{{\mathrm{T}}}}$ denote the random vector of the log HRs between exposure *e* and the $d = 1,2, \ldots ,80$ diseases, let ***β***_e_ denote the estimated vector of Cox model coefficients (log HRs) for the 80 diseases, let Σ_*e*_ denote the covariance matrix of those coefficients, where the diagonal represents the standard errors of the coefficients. The coefficients follow a multivariate normal distribution:

$${{{\boldsymbol{b}}}}_e\sim {{{\mathcal{N}}}}\left( {{{{\mathbf{\upbeta }}}}_e,{{{\mathbf{{\Sigma}}}}}_e} \right)$$

We can express the covariance matrix Σ_*e*_ in terms of the diagonal matrix ***D***_*e*_ = diag (**σ**_*e*_) that has the standard errors of the coefficients $\sigma _e = (\sigma _{e,1}, \ldots ,\sigma _{e,80})^T$ on the diagonal and the correlation matrix of the coefficients ***C*** as:

$${\Sigma}_e = {{{\boldsymbol{D}}}}_e{{{\boldsymbol{CD}}}}_e$$

so that

$${{{\boldsymbol{b}}}}_e\sim {{{\mathcal{N}}}}\left( {{{{\mathbf{\upbeta }}}}_e,{{{\boldsymbol{D}}}}_e{{{\boldsymbol{CD}}}}_e} \right)$$

We estimate ***β***_*e*_ and ***σ***_*e*_ from the 80 Cox models for each disease (for common variants we use the meta-analysis estimates from FinnGen and UKB). Let $d = 1,2, \ldots ,80$ index all the different diseases. We estimated ***C***_*d* × *d*_ by taking all the shrunk log HRs (assuming that they represent null effects) between all common variant–disease pairs and calculating the Pearson’s correlation coefficient between the log HRs of two diseases:

$$\widehat {{{\boldsymbol{C}}}}_{i,j} = {{{\boldsymbol{r}}}}(\widehat {{{\mathbf{\upbeta }}}}_i,\widehat {{{\mathbf{\upbeta }}}}_j)$$

where $\widehat {{{\mathbf{\upbeta }}}}_i$ and $\widehat {{{\mathbf{\upbeta }}}}_j$ are the Cox model coefficients for disease $i,j = 1,2, \ldots ,80$ for common variants not shrunk for diseases _*i*_, _*j*_ (at most 1,044). We restricted the correlation estimation to unshrunk variants to to make the coefficients reflect sampling variability, not true effects.

For each genetic exposure *e* we then resample the vector of log HRs $B = 1,2, \ldots ,10,000$ times from the multivariate normal distribution

$${{{\boldsymbol{b}}}}_{e,B}^ \ast \sim {{{\mathcal{N}}}}\left( {\widehat {{{\mathbf{\upbeta }}}}_{{{\boldsymbol{e}}}},\widehat {{{\boldsymbol{D}}}}_e\widehat {{{\boldsymbol{C}}}}\widehat {{{\boldsymbol{D}}}}_e} \right)$$

to emulate the sampling distribution of the vector of log HRs across all diseases that accounts for dependence in log HRs between diseases. We then repeat the estimation procedure for individual and population total attributable DALYs for each genetic exposure 10,000 times using the resampled ${{{\boldsymbol{b}}}}_{e,B}^ \ast$ to calculate the HRs instead of the maximum partial likelihood estimates from the Cox model ($\widehat \beta _i$). We then use the 2.5% and 97.5% percentiles of the resampled distribution as estimates of the 95% CIs and estimate the *P* values via a normal approximation.

#### FinnGen ethics statement

Patients and control participants in FinnGen provided informed consent for biobank research, based on the Finnish Biobank Act. Alternatively, separate research cohorts, collected before the Finnish Biobank Act came into effect (in September 2013) and start of FinnGen (August 2017), were collected based on study-specific consents and later transferred to the Finnish biobanks after approval by Fimea (Finnish Medicines Agency), the National Supervisory Authority for Welfare and Health. Recruitment protocols followed the biobank protocols approved by Fimea. The Coordinating Ethics Committee of the Hospital District of Helsinki and Uusimaa (HUS) statement number for the FinnGen study is HUS/990/2017.

The FinnGen study is approved by the Finnish Institute for Health and Welfare (permit nos. THL/2031/6.02.00/2017, THL/1101/5.05.00/2017, THL/341/6.02.00/2018, THL/2222/6.02.00/2018, THL/283/6.02.00/2019, THL/1721/5.05.00/2019 and THL/1524/5.05.00/2020), Digital and Population Data Service Agency (permit nos. VRK43431/2017-3, VRK/6909/2018-3 and VRK/4415/2019-3), the Social Insurance Institution (permit nos. KELA 58/522/2017, KELA 131/522/2018, KELA 70/522/2019, KELA 98/522/2019, KELA 134/522/2019, KELA 138/522/2019, KELA 2/522/2020 and KELA 16/522/2020), Findata permit nos. THL/2364/14.02/2020, THL/4055/14.06.00/2020, THL/3433/14.06.00/2020, THL/4432/14.06/2020, THL/5189/14.06/2020, THL/5894/14.06.00/2020, THL/6619/14.06.00/2020, THL/209/14.06.00/2021, THL/688/14.06.00/2021, THL/1284/14.06.00/2021, THL/1965/14.06.00/2021 and THL/5546/14.02.00/2020 and Statistics Finland (permit nos. TK-53-1041-17 and TK/143/07.03.00/2020 (earlier TK-53-90-20)).

The Biobank Access Decisions for FinnGen samples and data utilized in FinnGen Data Freeze 7 include: THL Biobank BB2017_55, BB2017_111, BB2018_19, BB_2018_34, BB_2018_67, BB2018_71, BB2019_7, BB2019_8, BB2019_26 and BB2020_1, Finnish Red Cross Blood Service Biobank 7.12.2017, Helsinki Biobank HUS/359/2017, Auria Biobank AB17-5154 and amendment no. 1 (17 August 2020), Biobank Borealis of Northern Finland_2017_1013, Biobank of Eastern Finland 1186/2018 and amendment 22 §/2020, Finnish Clinical Biobank Tampere MH0004 and amendments (21 February 2020 and 6 October 2020), Central Finland Biobank 1-2017 and Terveystalo Biobank STB 2018001.

#### UK Biobank ethics statement

UKB obtained ethics approval from the North West Multicentre Research Ethics Committee, which covers the United Kingdom (approval no. 11/NW/0382) and obtained informed consent from all participants. Our analyses were conducted under the UKB application no. 31063.

### Reporting summary

Further information on research design is available in the Nature Research Reporting Summary linked to this article.

## Online content

Any methods, additional references, Nature Research reporting summaries, source data, extended data, supplementary information, acknowledgements, peer review information; details of author contributions and competing interests; and statements of data and code availability are available at 10.1038/s41591-022-01957-2.

## Supplementary information

Supplementary Information FinnGen contributors

Reporting Summary

Supplementary Tables Supplementary Tables 1−16

## Data Availability

We present meta-analyzed HR estimates and all attributable DALY results in Supplementary Tables 5, 6, 8, 9 and 11−14. Results for common variants, HLA alleles and PGSs can additionally be explored through plots at https://dsge-lab.shinyapps.io/daly_genetics/. Individual-level genotypes and register data from FinnGen participants can be accessed by approved researchers via the Fingenious portal (https://site.fingenious.fi/en/) hosted by the Finnish Biobank Cooperative FinBB (https://finbb.fi/en/). Data release to FinBB is timed to the biannual public release of FinnGen summary results, which occurs 12 months after FinnGen consortium members can start working with the data. UKB data are available to approved researchers upon application (https://www.ukbiobank.ac.uk/enable-your-research/apply-for-access). Some of the datasets used in this study can be accessed in ClinVar (https://www.ncbi.nlm.nih.gov/clinvar/) and gnomAD (https://gnomad.broadinstitute.org/).

## Code availability

Custom code for the central parts of analyses can be found at https://github.com/dsgelab/dalys_code.
