# Supplementary material for: Genetic risk factors have a substantial impact on healthy life years
Source: Nat Med. 2022 Sep 12;28(9):1893–901. doi: 10.1038/s41591-022-01957-2 (PMC9499866; doi:10.1038/s41591-022-01957-2)
Supplement: Supplementary file 1 — FinnGen contributors [file 41591_2022_1957_MOESM1_ESM.pdf]

nature portfolio

https://doi.org/10.1038/s41591-022-01957-2

Supplementary information

Genetic risk factors have a substantial impact on healthy life years

In the format provided by the
authors and unedited

FinnGen contributors

Steering Committee

Aarno Palotie Institute for Molecular Medicine Finland, HiLIFE, University of Helsinki, Finland

Mark Daly Institute for Molecular Medicine Finland, HiLIFE, University of Helsinki, Finland

Pharmaceutical companies

Bridget Riley-Gills Abbvie, Chicago, IL, United States

Howard Jacob Abbvie, Chicago, IL, United States

Dirk Paul Astra Zeneca, Cambridge, United Kingdom

Heiko Runz Biogen, Cambridge, MA, United States

Sally John Biogen, Cambridge, MA, United States

George Okafo Boehringer Ingelheim, Ingelheim am Rhein, Germany

Nathan Lawless Boehringer Ingelheim, Ingelheim am Rhein, Germany

Robert Plenge Celgene, Summit, NJ, United States/Bristol Myers Squibb, New York, NY, United States

Joseph Maranville Celgene, Summit, NJ, United States/Bristol Myers Squibb, New York, NY, United States

Mark McCarthy Genentech, San Francisco, CA, United States

Julie Hunkapiller Genentech, San Francisco, CA, United States

Meg Ehm GlaxoSmithKline, Brentford, United Kingdom

Kirsi Auro GlaxoSmithKline, Brentford, United Kingdom

Simonne Longerich Merck, Kenilworth, NJ, United States

Caroline Fox Merck, Kenilworth, NJ, United States

Anders Mälarstig Pfizer, New York, NY, United States

Katherine Klinger Sanofi, Paris, France

Deepak Raipal Sanofi, Paris, France

Eric Green Maze Therapeutics, San Francisco, CA, United States

Robert Graham Maze Therapeutics, San Francisco, CA, United States

Robert Yang Janssen Biotech, Beerse, Belgium

Chris O´Donnell Novartis, Basel, Switzerland

University of Helsinki & Biobanks

Tomi Mäkelä HiLIFE, University of Helsinki, Finland, Finland

Jaakko Kaprio Institute for Molecular Medicine Finland, HiLIFE, Helsinki, Finland, Finland

Petri Virolainen Auria Biobank / University of Turku / Hospital District of Southwest Finland, Turku, Finland

Antti Hakanen Auria Biobank / University of Turku / Hospital District of Southwest Finland, Turku, Finland

Terhi Kilpi THL Biobank / The National Institute of Health and Welfare Helsinki, Finland

Markus Perola THL Biobank / The National Institute of Health and Welfare Helsinki, Finland

Jukka Partanen Finnish Red Cross Blood Service / Finnish Hematology Registry and Clinical Biobank, Helsinki, Finland

Anne Pitkäranta Helsinki Biobank / Helsinki University and Hospital District of Helsinki and Uusimaa, Helsinki

Juhani Junttila Northern Finland Biobank Borealis / University of Oulu / Northern Ostrobothnia Hospital District, Oulu, Finland

Raisa Serpi Northern Finland Biobank Borealis / University of Oulu / Northern Ostrobothnia Hospital District, Oulu, Finland

Tarja Laitinen Finnish Clinical Biobank Tampere **/** University of Tampere / Pirkanmaa Hospital District, Tampere, Finland

Veli-Matti Kosma Biobank of Eastern Finland / University of Eastern Finland / Northern Savo Hospital District, Kuopio, Finland

Jari Laukkanen Central Finland Biobank / University of Jyväskylä / Central Finland Health Care District, Jyväskylä, Finland

Marco Hautalahti FINBB - Finnish biobank cooperative

Other Experts/ Non-Voting Members

Outi Tuovila Business Finland, Helsinki, Finland

Raimo Pakkanen Business Finland, Helsinki, Finland

Scientific Committee

Pharmaceutical companies

Jeffrey Waring	Abbvie, Chicago, IL, United States
Bridget Riley-Gillis	Abbvie, Chicago, IL, United States
Ioanna Tachmazidou	Astra Zeneca, Cambridge, United Kingdom
Chia-Yen Chen	Biogen, Cambridge, MA, United States
Heiko Runz	Biogen, Cambridge, MA, United States
Zhihao Ding	Boehringer Ingelheim, Ingelheim am Rhein, Germany
Marc Jung	Boehringer Ingelheim, Ingelheim am Rhein, Germany
Shameek Biswas	Celgene, Summit, NJ, United States/Bristol Myers Squibb, New York, NY, United States
Rion Pendergrass	Genentech, San Francisco, CA, United States
Julie Hunkapiller	Genentech, San Francisco, CA, United States
Meg Ehm	GlaxoSmithKline, Brentford, United Kingdom
David Pulford	GlaxoSmithKline, Brentford, United Kingdom
Neha Raghavan	Merck, Kenilworth, NJ, United States
Adriana Huertas-Vazquez	Merck, Kenilworth, NJ, United States
Jae-Hoon Sul	Merck, Kenilworth, NJ, United States
Anders Mälarstig	Pfizer, New York, NY, United States
Xinli Hu	Pfizer, New York, NY, United States
Katherine Klinger	Sanofi, Paris, France
Matthias Gossel	Sanofi, Paris, France
Robert Graham	Maze Therapeutics, San Francisco, CA, United States
Eric Green	Maze Therapeutics, San Francisco, CA, United States
Sahar Mozaffari	Maze Therapeutics, San Francisco, CA, United States
Dawn Waterworth	Janssen Research & Development, LLC, Spring House, PA, United States

Nicole Renaud Novartis, Basel, Switzerland

Ma´en Obeidat Novartis, Basel, Switzerland

University of Helsinki & Biobanks

Samuli Ripatti Institute for Molecular Medicine Finland, HiLIFE, Helsinki, Finland

Johanna Schleutker Auria Biobank / Univ. of Turku / Hospital District of Southwest Finland, Turku, Finland

Markus Perola THL Biobank / The National Institute of Health and Welfare Helsinki, Finland

Mikko Arvas Finnish Red Cross Blood Service / Finnish Hematology Registry and Clinical Biobank, Helsinki, Finland

Olli Carpén Helsinki Biobank / Helsinki University and Hospital District of Helsinki and Uusimaa, Helsinki

Reetta Hinttala Northern Finland Biobank Borealis / University of Oulu / Northern Ostrobothnia Hospital District, Oulu, Finland

Johannes Kettunen Northern Finland Biobank Borealis / University of Oulu / Northern Ostrobothnia Hospital District, Oulu, Finland

Arto Mannermaa Biobank of Eastern Finland / University of Eastern Finland / Northern Savo Hospital District, Kuopio, Finland

Katriina Aalto-Setälä Finnish Clinical Biobank Tampere / University of Tampere / Pirkanmaa Hospital District, Tampere, Finland

Mika Kähönen Finnish Clinical Biobank Tampere / University of Tampere / Pirkanmaa Hospital District, Tampere, Finland

Jari Laukkanen Central Finland Biobank / University of Jyväskylä / Central Finland Health Care District, Jyväskylä, Finland

Johanna Mäkelä FINBB - Finnish biobank cooperative

Clinical Groups

Neurology Group

Reetta Kälviäinen Northern Savo Hospital District, Kuopio, Finland

Valtteri Julkunen Northern Savo Hospital District, Kuopio, Finland

Hilkka Soininen Northern Savo Hospital District, Kuopio, Finland

Anne Remes	Northern Ostrobothnia Hospital District, Oulu, Finland
Mikko Hiltunen	Northern Savo Hospital District, Kuopio, Finland
Jukka Peltola	Pirkanmaa Hospital District, Tampere, Finland
Minna Raivio	Hospital District of Helsinki and Uusimaa, Helsinki, Finland
Pentti Tienari	Hospital District of Helsinki and Uusimaa, Helsinki, Finland
Juha Rinne	Hospital District of Southwest Finland, Turku, Finland
Roosa Kallionpää	Hospital District of Southwest Finland, Turku, Finland
Juulia Partanen	Institute for Molecular Medicine Finland, HiLIFE, University of Helsinki, Finland
Ali Abbasi	Abbvie, Chicago, IL, United States
Adam Ziemann	Abbvie, Chicago, IL, United States
Jeffrey Waring	Abbvie, Chicago, IL, United States
Nizar Smaoui	Abbvie, Chicago, IL, United States
Anne Lehtonen	Abbvie, Chicago, IL, United States
Susan Eaton	Biogen, Cambridge, MA, United States
Heiko Runz	Biogen, Cambridge, MA, United States
Sanni Lahdenperä	Biogen, Cambridge, MA, United States
Janet van Adelsberg	Celgene, Summit, NJ, United States/ Bristol Myers Squibb, New York, NY, United States
Shameek Biswas	Celgene, Summit, NJ, United States/ Bristol Myers Squibb, New York, NY, United States
Julie Hunkapiller	Genentech, San Francisco, CA, United States
Natalie Bowers	Genentech, San Francisco, CA, United States
Edmond Teng	Genentech, San Francisco, CA, United States
Rion Pendergrass	Genentech, San Francisco, CA, United States
Fanli Xu	GlaxoSmithKline, Brentford, United Kingdom
David Pulford	GlaxoSmithKline, Brentford, United Kingdom
Kirsi Auro	GlaxoSmithKline, Brentford, United Kingdom
Laura Addis	GlaxoSmithKline, Brentford, United Kingdom

John Eicher GlaxoSmithKline, Brentford, United Kingdom

Qingqin S Li Janssen Research & Development, LLC, Titusville, NJ 08560, United States

Karen He Janssen Research & Development, LLC, Spring House, PA, United States

Ekaterina Khramtsova Janssen Research & Development, LLC, Spring House, PA, United States

Neha Raghavan Merck, Kenilworth, NJ, United States

Kari Linden Pfizer, New York, NY, United States

Gastroenterology Group

Martti Färkkilä Hospital District of Helsinki and Uusimaa, Helsinki, Finland

Jukka Koskela Hospital District of Helsinki and Uusimaa, Helsinki, Finland

Sampsa Pikkarainen Hospital District of Helsinki and Uusimaa, Helsinki, Finland

Airi Jussila Pirkanmaa Hospital District, Tampere, Finland

Katri Kaukinen Pirkanmaa Hospital District, Tampere, Finland

Timo Blomster Northern Ostrobothnia Hospital District, Oulu, Finland

Mikko Kiviniemi Northern Savo Hospital District, Kuopio, Finland

Markku Voutilainen Hospital District of Southwest Finland, Turku, Finland

Mark Daly Institute for Molecular Medicine Finland, HiLIFE, University of Helsinki, Finland

Ali Abbasi Abbvie, Chicago, IL, United States

Graham Heap Abbvie, Chicago, IL, United States

Jeffrey Waring Abbvie, Chicago, IL, United States

Nizar Smaoui Abbvie, Chicago, IL, United States

Fedik Rahimov Abbvie, Chicago, IL, United States

Anne Lehtonen Abbvie, Chicago, IL, United States

Keith Usiskin Celgene, Summit, NJ, United States/ Bristol Myers Squibb, New York, NY, United States

Tim Lu Genentech, San Francisco, CA, United States

Natalie Bowers Genentech, San Francisco, CA, United States

Rion Pendergrass Genentech, San Francisco, CA, United States

Linda McCarthy GlaxoSmithKline, Brentford, United Kingdom

Amy Hart Janssen Research & Development, LLC, Spring House, PA, United States

Meijian Guan Janssen Research & Development, LLC, Spring House, PA, United States

Jason Miller Merck, Kenilworth, NJ, United States

Kirsi Kalpala Pfizer, New York, NY, United States

Melissa Miller Pfizer, New York, NY, United States

Xinli Hu Pfizer, New York, NY, United States

Rheumatology Group

Kari Eklund Hospital District of Helsinki and Uusimaa, Helsinki, Finland

Antti Palomäki Hospital District of Southwest Finland, Turku, Finland

Pia Isomäki Pirkanmaa Hospital District, Tampere, Finland

Laura Pirilä Hospital District of Southwest Finland, Turku, Finland

Oili Kaipiainen-Seppänen Northern Savo Hospital District, Kuopio, Finland

Johanna Huhtakangas Northern Ostrobothnia Hospital District, Oulu, Finland

Nina Mars Institute for Molecular Medicine Finland, HiLIFE, Helsinki, Finland

Ali Abbasi Abbvie, Chicago, IL, United States

Jeffrey Waring Abbvie, Chicago, IL, United States

Fedik Rahimov Abbvie, Chicago, IL, United States

Apinya Lertratanakul Abbvie, Chicago, IL, United States

Nizar Smaoui Abbvie, Chicago, IL, United States

Anne Lehtonen Abbvie, Chicago, IL, United States

David Close Astra Zeneca, Cambridge, United Kingdom

Marla Hochfeld Celgene, Summit, NJ, United States/ Bristol Myers Squibb, New York, NY, United States

Natalie Bowers Genentech, San Francisco, CA, United States

Rion Pendergrass Genentech, San Francisco, CA, United States

Jorge Esparza Gordillo GlaxoSmithKline, Brentford, United Kingdom

Kirsi Auro GlaxoSmithKline, Brentford, United Kingdom

Dawn Waterworth Janssen Research & Development, LLC, Spring House, PA, United States

Fabiana Farias Merck, Kenilworth, NJ, United States

Kirsi Kalpala Pfizer, New York, NY, United States

Nan Bing Pfizer, New York, NY, United States

Xinli Hu Pfizer, New York, NY, United States

Pulmonology Group

Tarja Laitinen Pirkanmaa Hospital District, Tampere, Finland

Margit Pelkonen Northern Savo Hospital District, Kuopio, Finland

Paula Kauppi Hospital District of Helsinki and Uusimaa, Helsinki, Finland

Hannu Kankaanranta University of Gothenburg, Gothenburg, Sweden/ Seinäjoki Central Hospital, Seinäjoki, Finland/ Tampere University, Tampere, Finland

Terttu Harju Northern Ostrobothnia Hospital District, Oulu, Finland

Riitta Lahesmaa Hospital District of Southwest Finland, Turku, Finland

Nizar Smaoui Abbvie, Chicago, IL, United States

Alex Mackay Astra Zeneca, Cambridge, United Kingdom

Glenda Lassi Astra Zeneca, Cambridge, United Kingdom

Susan Eaton Biogen, Cambridge, MA, United States

Steven Greenberg Celgene, Summit, NJ, United States/ Bristol Myers Squibb, New York, NY, United States

Hubert Chen Genentech, San Francisco, CA, United States

Rion Pendergrass Genentech, San Francisco, CA, United States

Natalie Bowers Genentech, San Francisco, CA, United States

Joanna Betts GlaxoSmithKline, Brentford, United Kingdom

Kirsi Auro GlaxoSmithKline, Brentford, United Kingdom

Rajashree Mishra	GlaxoSmithKline, Brentford, United Kingdom
Majd Mouded	Novartis, Basel, Switzerland
Debby Ngo	Novartis, Basel, Switzerland

Cardiometabolic Diseases Group

Teemu Niiranen	The National Institute of Health and Welfare Helsinki, Finland
Felix Vaura	The National Institute of Health and Welfare Helsinki, Finland
Veikko Salomaa	The National Institute of Health and Welfare Helsinki, Finland
Kaj Metsärinne	Hospital District of Southwest Finland, Turku, Finland
Jenni Aittokallio	Hospital District of Southwest Finland, Turku, Finland
Mika Kähönen	Pirkanmaa Hospital District, Tampere, Finland
Jussi Hernesniemi	Pirkanmaa Hospital District, Tampere, Finland
Juhani Junttila	Northern Ostrobothnia Hospital District, Oulu, Finland
Markku Laakso	Northern Savo Hospital District, Kuopio, Finland
Jussi Pihlajamäki	Northern Savo Hospital District, Kuopio, Finland
Daniel Gordin	Hospital District of Helsinki and Uusimaa, Helsinki, Finland
Juha Sinisalo	Hospital District of Helsinki and Uusimaa, Helsinki, Finland
Marja-Riitta Taskinen	Hospital District of Helsinki and Uusimaa, Helsinki, Finland
Tiinamaija Tuomi	Hospital District of Helsinki and Uusimaa, Helsinki, Finland
Timo Hiltunen	Hospital District of Helsinki and Uusimaa, Helsinki, Finland
Jari Laukkanen	Central Finland Health Care District, Jyväskylä, Finland
Amanda Elliott	Institute for Molecular Medicine Finland, HiLIFE, University of Helsinki, Finland / Broad Institute, Cambridge, MA, United States
Mary Pat Reeve	Institute for Molecular Medicine Finland, HiLIFE, University of Helsinki, Finland
Sanni Ruotsalainen	Institute for Molecular Medicine Finland, HiLIFE, University of Helsinki, Finland
Benjamin Challis	Astra Zeneca, Cambridge, United Kingdom
Dirk Paul	Astra Zeneca, Cambridge, United Kingdom

Keith Usiskin	Celgene, Summit, NJ, United States/ Bristol Myers Squibb, New York, NY, United States
Julie Hunkapiller	Genentech, San Francisco, CA, United States
Natalie Bowers	Genentech, San Francisco, CA, United States
Rion Pendergrass	Genentech, San Francisco, CA, United States
Audrey Chu	GlaxoSmithKline, Brentford, United Kingdom
Kirsi Auro	GlaxoSmithKline, Brentford, United Kingdom
Dermot Reilly	Janssen Research & Development, LLC, Boston, MA, United States
Mike Mendelson	Novartis, Boston, MA, United States
Jaakko Parkkinen	Pfizer, New York, NY, United States
Melissa Miller	Pfizer, New York, NY, United States

Oncology Group

Tuomo Meretoja	Hospital District of Helsinki and Uusimaa, Helsinki, Finland
Heikki Joensuu	Hospital District of Helsinki and Uusimaa, Helsinki, Finland
Olli Carpén	Hospital District of Helsinki and Uusimaa, Helsinki, Finland
Lauri Aaltonen	Hospital District of Helsinki and Uusimaa, Helsinki, Finland
Johanna Mattson	Hospital District of Helsinki and Uusimaa, Helsinki, Finland
Eveliina Salminen	Hospital District of Helsinki and Uusimaa, Helsinki, Finland
Annika Auranen	Pirkanmaa Hospital District , Tampere, Finland
Peeter Karihtala	Northern Ostrobothnia Hospital District, Oulu, Finland
Päivi Auvinen	Northern Savo Hospital District, Kuopio, Finland
Klaus Elenius	Hospital District of Southwest Finland, Turku, Finland
Johanna Schleutker	Hospital District of Southwest Finland, Turku, Finland
Esa Pitkänen	Institute for Molecular Medicine Finland, HiLIFE, University of Helsinki, Finland
Nina Mars	Institute for Molecular Medicine Finland, HiLIFE, University of Helsinki, Finland
Mark Daly	Institute for Molecular Medicine Finland, HiLIFE, University of Helsinki, Finland
Relja Popovic	Abbvie, Chicago, IL, United States

Jeffrey Waring	Abbvie, Chicago, IL, United States
Bridget Riley-Gillis	Abbvie, Chicago, IL, United States
Anne Lehtonen	Abbvie, Chicago, IL, United States
Jennifer Schutzman	Genentech, San Francisco, CA, United States
Julie Hunkapiller	Genentech, San Francisco, CA, United States
Natalie Bowers	Genentech, San Francisco, CA, United States
Rion Pendergrass	Genentech, San Francisco, CA, United States
Diptee Kulkarni	GlaxoSmithKline, Brentford, United Kingdom
Kirsi Auro	GlaxoSmithKline, Brentford, United Kingdom
Alessandro Porello	Janssen Research & Development, LLC, Spring House, PA, United States
Andrey Loboda	Merck, Kenilworth, NJ, United States
Heli Lehtonen	Pfizer, New York, NY, United States
Stefan McDonough	Pfizer, New York, NY, United States
Marika Crohns	Sanofi, Paris, France
Sauli Vuoti	Sanofi, Paris, France

Opthalmology Group

Kai Kaarniranta	Northern Savo Hospital District, Kuopio, Finland
Joni A Turunen	Hospital District of Helsinki and Uusimaa, Helsinki, Finland
Terhi Ollila	Hospital District of Helsinki and Uusimaa, Helsinki, Finland
Hannu Uusitalo	Pirkanmaa Hospital District, Tampere, Finland
Juha Karjalainen	Institute for Molecular Medicine Finland, HiLIFE, University of Helsinki, Finland
Esa Pitkänen	Institute for Molecular Medicine Finland, HiLIFE, University of Helsinki, Finland

Mengzhen Liu	Abbvie, Chicago, IL, United States
Heiko Runz	Biogen, Cambridge, MA, United States
Stephanie Loomis	Biogen, Cambridge, MA, United States
Erich Strauss	Genentech, San Francisco, CA, United States

Natalie Bowers Genentech, San Francisco, CA, United States

Hao Chen Genentech, San Francisco, CA, United States

Rion Pendergrass Genentech, San Francisco, CA, United States

Dermatology Group

Kaisa Tasanen Northern Ostrobothnia Hospital District, Oulu, Finland

Laura Huilaja Northern Ostrobothnia Hospital District, Oulu, Finland

Katariina Hannula-Jouppi Hospital District of Helsinki and Uusimaa, Helsinki, Finland

Teea Salmi Pirkanmaa Hospital District, Tampere, Finland

Sirkku Peltonen Hospital District of Southwest Finland, Turku, Finland

Leena Koulu Hospital District of Southwest Finland, Turku, Finland

Nizar Smaoui Abbvie, Chicago, IL, United States

Fedik Rahimov Abbvie, Chicago, IL, United States

Anne Lehtonen Abbvie, Chicago, IL, United States

David Choy Genentech, San Francisco, CA, United States

Rion Pendergrass Genentech, San Francisco, CA, United States

Dawn Waterworth Janssen Research & Development, LLC, Spring House, PA, United States

Kirsi Kalpala Pfizer, New York, NY, United States

Ying Wu Pfizer, New York, NY, United States

Odontology Group

Pirkko Pussinen Hospital District of Helsinki and Uusimaa, Helsinki, Finland

Aino Salminen Hospital District of Helsinki and Uusimaa, Helsinki, Finland

Tuula Salo Hospital District of Helsinki and Uusimaa, Helsinki, Finland

David Rice Hospital District of Helsinki and Uusimaa, Helsinki, Finland

Pekka Nieminen Hospital District of Helsinki and Uusimaa, Helsinki, Finland

Ulla Palotie Hospital District of Helsinki and Uusimaa, Helsinki, Finland

Maria Siponen Northern Savo Hospital District, Kuopio, Finland

Liisa Suominen	Northern Savo Hospital District, Kuopio, Finland
Päivi Mäntylä	Northern Savo Hospital District, Kuopio, Finland
Ulvi Gursoy	Hospital District of Southwest Finland, Turku, Finland
Vuokko Anttonen	Northern Ostrobothnia Hospital District, Oulu, Finland
Kirsi Sipilä	Northern Ostrobothnia Hospital District, Oulu, Finland
Rion Pendergrass	Genentech, San Francisco, CA, United States

Women's Health and Reproduction Group

Hannele Laivuori	Institute for Molecular Medicine Finland, HiLIFE, University of Helsinki, Finland
Venla Kurra	Pirkanmaa Hospital District, Tampere, Finland
Laura Kotaniemi-Talonen	Pirkanmaa Hospital District, Tampere, Finland
Oskari Heikinheimo	Hospital District of Helsinki and Uusimaa, Helsinki, Finland
Ilkka Kalliala	Hospital District of Helsinki and Uusimaa, Helsinki, Finland
Lauri Aaltonen	Hospital District of Helsinki and Uusimaa, Helsinki, Finland
Varpu Jokimaa	Hospital District of Southwest Finland, Turku, Finland
Johannes Kettunen	Northern Ostrobothnia Hospital District, Oulu, Finland
Marja Vääräsmäki	Northern Ostrobothnia Hospital District, Oulu, Finland
Outi Uimari	Northern Ostrobothnia Hospital District, Oulu, Finland
Laure Morin-Papunen	Northern Ostrobothnia Hospital District, Oulu, Finland
Maarit Niinimäki	Northern Ostrobothnia Hospital District, Oulu, Finland
Terhi Piltonen	Northern Ostrobothnia Hospital District, Oulu, Finland
Katja Kivinen	Institute for Molecular Medicine Finland, HiLIFE, University of Helsinki, Finland
Elisabeth Widen	Institute for Molecular Medicine Finland, HiLIFE, University of Helsinki, Finland
Taru Tukiainen	Institute for Molecular Medicine Finland, HiLIFE, University of Helsinki, Finland
Mary Pat Reeve	Institute for Molecular Medicine Finland, HiLIFE, University of Helsinki, Finland
Mark Daly	Institute for Molecular Medicine Finland, HiLIFE, University of Helsinki, Finland
Liu Aoxing	Institute for Molecular Medicine Finland, HiLIFE, University of Helsinki, Finland
Andrea Ganna	Institute for Molecular Medicine Finland, HiLIFE, University of Helsinki, Finland

Niko Välimäki	University of Helsinki, Helsinki, Finland
Eija Laakkonen	University of Jyväskylä, Jyväskylä, Finland
Jaakko Tyrmi	University of Oulu, Oulu, Finland / University of Tampere, Tampere, Finland
Heidi Silven	University of Oulu, Oulu, Finland
Eeva Slitz	University of Oulu, Oulu, Finland
Riikka Arffman	University of Oulu, Oulu, Finland
Susanna Savukoski	University of Oulu, Oulu, Finland
Triin Laisk	Estonian biobank, Tartu, Estonia
Natalia Pujol	Estonian biobank, Tartu, Estonia
Bridget Riley-Gillis	Abbvie, Chicago, IL, United States
Mengzhen Liu	Abbvie, Chicago, IL, United States
Rion Pendergrass	Genentech, San Francisco, CA, United States
Janet Kumar	GlaxoSmithKline, Brentford, United Kingdom
Kirsi Auro	GlaxoSmithKline, Brentford, United Kingdom

FinnGen Analysis working group

Bridget Riley-Gillis	Abbvie, Chicago, IL, United States
Reza Hammond	Abbvie, Chicago, IL, United States
Fedik Rahimov	Abbvie, Chicago, IL, United States
Sabah Kadri	Abbvie, Chicago, IL, United States
Mengzhen Liu	Abbvie, Chicago, IL, United States
Slavé Petrovski	Astra Zeneca, Cambridge, United Kingdom
Eleonor Wigmore	Astra Zeneca, Cambridge, United Kingdom
Adele Mitchell	Biogen, Cambridge, MA, United States
Benjamin Sun	Biogen, Cambridge, MA, United States
Ellen Tsai	Biogen, Cambridge, MA, United States
Denis Baird	Biogen, Cambridge, MA, United States
Paola Bronson	Biogen, Cambridge, MA, United States

Ruoyu Tian	Biogen, Cambridge, MA, United States
Stephanie Loomis	Biogen, Cambridge, MA, United States
Yunfeng Huang	Biogen, Cambridge, MA, United States
Till Andlauer	Boehringer Ingelheim, Ingelheim am Rhein, Germany
Jatin Arora	Boehringer Ingelheim, Ingelheim am Rhein, Germany
Ghadi Rai	Boehringer Ingelheim, Ingelheim am Rhein, Germany
Zhihao Ding	Boehringer Ingelheim, Ingelheim am Rhein, Germany
Lorenz Maier	Boehringer Ingelheim, Ingelheim am Rhein, Germany
Karsten Quast	Boehringer Ingelheim, Ingelheim am Rhein, Germany
Francisco Herruzo	Boehringer Ingelheim, Ingelheim am Rhein, Germany
Daniel Lopez	Boehringer Ingelheim, Ingelheim am Rhein, Germany
Marc Jung	Boehringer Ingelheim, Ingelheim am Rhein, Germany
Boris Bartholdy	Boehringer Ingelheim, Ingelheim am Rhein, Germany
Joseph Maranville	Celgene, Summit, NJ, United States/ Bristol Myers Squibb, New York, NY, United States
Shameek Biswas	Celgene, Summit, NJ, United States/ Bristol Myers Squibb, New York, NY, United States
Elmutaz Mohammed	Celgene, Summit, NJ, United States/ Bristol Myers Squibb, New York, NY, United States
Samir Wadhawan	Celgene, Summit, NJ, United States/ Bristol Myers Squibb, New York, NY, United States
Erika Kvikstad	Celgene, Summit, NJ, United States/ Bristol Myers Squibb, New York, NY, United States
Diana Chang	Genentech, San Francisco, CA, United States
Julie Hunkapiller	Genentech, San Francisco, CA, United States
Tushar Bhangale	Genentech, San Francisco, CA, United States
Natalie Bowers	Genentech, San Francisco, CA, United States
Rion Pendergrass	Genentech, San Francisco, CA, United States
Karen S King	GlaxoSmithKline, Brentford, United Kingdom

Padhraig Gormley	GlaxoSmithKline, Brentford, United Kingdom
Jimmy Liu	GlaxoSmithKline, Brentford, United Kingdom
Karsten Sieber	Janssen Research & Development, LLC, Spring House, PA, United States
Amy Hart	Janssen Research & Development, LLC, Spring House, PA, United States
Meijian Guan	Janssen Research & Development, LLC, Spring House, PA, United States
Shicheng Guo	Janssen Research & Development, LLC, Spring House, PA, United States
Matt Brauer	Maze Therapeutics, San Francisco, CA, United States
Jason Miller	Merck, Kenilworth, NJ, United States
Fabiana Farias	Merck, Kenilworth, NJ, United States
Jorge Del-Aguila	Merck, Kenilworth, NJ, United States
Kirill Shkura	Merck, Kenilworth, NJ, United States
Victor Neduva	Merck, Kenilworth, NJ, United States
Huilei Xu	Novartis, Basel, Switzerland
Amy Cole	Novartis, Basel, Switzerland
Jonathan Chung	Novartis, Basel, Switzerland
Jaison Jacob	Novartis, Basel, Switzerland
Katrina de Lange	Novartis, Basel, Switzerland
Jonas Zierer	Novartis, Basel, Switzerland
Xing Chen	Pfizer, New York, NY, United States
Åsa Hedman	Pfizer, New York, NY, United States
Franck Auge	Sanofi, Paris, France
Clement Chatelain	Sanofi, Paris, France
Deepak Rajpal	Sanofi, Paris, France
Dongyu Liu	Sanofi, Paris, France
Katherine Call	Sanofi, Paris, France
Tai-He Xia	Sanofi, Paris, France

Mitja Kurki Institute for Molecular Medicine Finland, HiLIFE, University of Helsinki, Finland / Broad Institute, Cambridge, MA, United States

Samuli Ripatti Institute for Molecular Medicine Finland, HiLIFE, University of Helsinki, Finland

Mark Daly Institute for Molecular Medicine Finland, HiLIFE, University of Helsinki, Finland

Juha Karjalainen Institute for Molecular Medicine Finland, HiLIFE, University of Helsinki, Finland

Aki Havulinna Institute for Molecular Medicine Finland, HiLIFE, University of Helsinki, Finland

Juha Mehtonen Institute for Molecular Medicine Finland, HiLIFE, University of Helsinki, Finland

Priit Palta Institute for Molecular Medicine Finland, HiLIFE, University of Helsinki, Finland

Shabbeer Hassan Institute for Molecular Medicine Finland, HiLIFE, University of Helsinki, Finland

Pietro Della Briotta Parolo Institute for Molecular Medicine Finland, HiLIFE, University of Helsinki, Finland

Wei Zhou Broad Institute, Cambridge, MA, United States

Mutaamba Maasha Broad Institute, Cambridge, MA, United States

Shabbeer Hassan Institute for Molecular Medicine Finland, HiLIFE, University of Helsinki, Finland

Susanna Lemmelä Institute for Molecular Medicine Finland, HiLIFE, University of Helsinki, Finland

Manuel Rivas University of Stanford, Stanford, CA, United States

Aarno Palotie Institute for Molecular Medicine Finland, HiLIFE, University of Helsinki, Finland

Arto Lehisto Institute for Molecular Medicine Finland, HiLIFE, University of Helsinki, Finland

Andrea Ganna Institute for Molecular Medicine Finland, HiLIFE, University of Helsinki, Finland

Vincent Llorens Institute for Molecular Medicine Finland, HiLIFE, University of Helsinki, Finland

Hannele Laivuori Institute for Molecular Medicine Finland, HiLIFE, University of Helsinki, Finland

Taru Tukiainen Institute for Molecular Medicine Finland, HiLIFE, University of Helsinki, Finland

Mary Pat Reeve Institute for Molecular Medicine Finland, HiLIFE, University of Helsinki, Finland

Henrike Heyne Institute for Molecular Medicine Finland, HiLIFE, University of Helsinki, Finland

Nina Mars Institute for Molecular Medicine Finland, HiLIFE, University of Helsinki, Finland

Kimmo Palin University of Helsinki, Helsinki, Finland

Javier Garcia-Tabuenca University of Tampere, Tampere, Finland

Harri Siirtola University of Tampere, Tampere, Finland

Tuomo Kiiskinen Institute for Molecular Medicine Finland, HiLIFE, University of Helsinki, Finland

Jiwoo Lee Institute for Molecular Medicine Finland, HiLIFE, University of Helsinki, Finland / Broad Institute, Cambridge, MA, United States

Kristin Tsuo Institute for Molecular Medicine Finland, HiLIFE, University of Helsinki, Finland / Broad Institute, Cambridge, MA, United States

Amanda Elliott Institute for Molecular Medicine Finland, HiLIFE, University of Helsinki, Finland / Broad Institute, Cambridge, MA, United States

Kati Kristiansson THL Biobank / The National Institute of Health and Welfare Helsinki, Finland

Mikko Arvas Finnish Red Cross Blood Service / Finnish Hematology Registry and Clinical Biobank, Helsinki, Finland

Kati Hyvärinen Finnish Red Cross Blood Service, Helsinki, Finland

Jarmo Ritari Finnish Red Cross Blood Service, Helsinki, Finland

Olli Carpén Helsinki Biobank / Helsinki University and Hospital District of Helsinki and Uusimaa, Helsinki

Johannes Kettunen Northern Finland Biobank Borealis / University of Oulu / Northern Ostrobothnia Hospital District, Oulu, Finland

Katri Pylkäs University of Oulu, Oulu, Finland

Eeva Sliz University of Oulu, Oulu, Finland

Minna Karjalainen University of Oulu, Oulu, Finland

Tuomo Mantere Northern Finland Biobank Borealis / University of Oulu / Northern Ostrobothnia Hospital District, Oulu, Finland

Eeva Kangasniemi Finnish Clinical Biobank Tampere / University of Tampere / Pirkanmaa Hospital District, Tampere, Finland

Sami Heikkinen University of Eastern Finland, Kuopio, Finland

Arto Mannermaa Biobank of Eastern Finland / University of Eastern Finland / Northern Savo Hospital District, Kuopio, Finland

Eija Laakkonen University of Jyväskylä, Jyväskylä, Finland

Dhanaprakash Jambulingam University of Turku, Turku, Finland

Venkat Subramaniam Rathinakannan University of Turku, Turku, Finland

Nina Pitkänen Auria Biobank / University of Turku / Hospital District of Southwest Finland, Turku, Finland

Biobank directors

Lila Kallio Auria Biobank / University of Turku / Hospital District of Southwest Finland, Turku, Finland

Sirpa Soini THL Biobank / The National Institute of Health and Welfare Helsinki, Finland

Jukka Partanen Finnish Red Cross Blood Service / Finnish Hematology Registry and Clinical Biobank, Helsinki, Finland

Eero Punkka Helsinki Biobank / Helsinki University and Hospital District of Helsinki and Uusimaa, Helsinki

Raisa Serpi Northern Finland Biobank Borealis / University of Oulu / Northern Ostrobothnia Hospital District, Oulu, Finland

Sanna Siltanen Finnish Clinical Biobank Tampere / University of Tampere / Pirkanmaa Hospital District, Tampere, Finland

Veli-Matti Kosma Biobank of Eastern Finland / University of Eastern Finland / Northern Savo Hospital District, Kuopio, Finland

Teijo Kuopio Central Finland Biobank / University of Jyväskylä / Central Finland Health Care District, Jyväskylä, Finland

FinnGen Teams

Administration

Anu Jalanko Institute for Molecular Medicine Finland, HiLIFE, University of Helsinki, Finland

Huei-Yi Shen Institute for Molecular Medicine Finland, HiLIFE, University of Helsinki, Finland

Risto Kajanne Institute for Molecular Medicine Finland, HiLIFE, University of Helsinki, Finland

Mervi Aavikko Institute for Molecular Medicine Finland, HiLIFE, University of Helsinki, Finland

Analysis

Mitja Kurki Institute for Molecular Medicine Finland, HiLIFE, University of Helsinki, Finland / Broad Institute, Cambridge, MA, United States

Juha Karjalainen Institute for Molecular Medicine Finland, HiLIFE, University of Helsinki, Finland

Pietro Della Briotta Parolo Institute for Molecular Medicine Finland, HiLIFE, University of Helsinki, Finland

Arto Lehisto Institute for Molecular Medicine Finland, HiLIFE, University of Helsinki, Finland

Juha Mehtonen Institute for Molecular Medicine Finland, HiLIFE, University of Helsinki, Finland

Wei Zhou Broad Institute, Cambridge, MA, United States

Masahiro Kanai Broad Institute, Cambridge, MA, United States

Mutaamba Maasha Broad Institute, Cambridge, MA, United States

Clinical Endpoint Development

Hannele Laivuori Institute for Molecular Medicine Finland, HiLIFE, University of Helsinki, Finland

Aki Havulinna Institute for Molecular Medicine Finland, HiLIFE, University of Helsinki, Finland

Susanna Lemmelä Institute for Molecular Medicine Finland, HiLIFE, University of Helsinki, Finland

Tuomo Kiiskinen Institute for Molecular Medicine Finland, HiLIFE, University of Helsinki, Finland

L. Elisa Lahtela Institute for Molecular Medicine Finland, HiLIFE, University of Helsinki, Finland

Communication

Mari Kaunisto Institute for Molecular Medicine Finland, HiLIFE, University of Helsinki, Finland

E-Science

Elina Kilpeläinen Institute for Molecular Medicine Finland, HiLIFE, University of Helsinki, Finland

Timo P. Sipilä Institute for Molecular Medicine Finland, HiLIFE, University of Helsinki, Finland

Oluwaseun Alexander Dada Institute for Molecular Medicine Finland, HiLIFE, University of Helsinki, Finland

Awaisa Ghazal Institute for Molecular Medicine Finland, HiLIFE, University of Helsinki, Finland

Anastasia Shcherban Institute for Molecular Medicine Finland, HiLIFE, University of Helsinki, Finland

Rigbe Weldatsadik Institute for Molecular Medicine Finland, HiLIFE, University of Helsinki, Finland

Genotyping

Kati Donner Institute for Molecular Medicine Finland, HiLIFE, University of Helsinki, Finland

Timo P. Sipilä Institute for Molecular Medicine Finland, HiLIFE, University of Helsinki, Finland

Sample Collection Coordination

Anu Loukola Helsinki Biobank / Helsinki University and Hospital District of Helsinki and Uusimaa, Helsinki

Sample Logistics

Päivi Laiho THL Biobank / The National Institute of Health and Welfare Helsinki, Finland

Tuuli Sistonen THL Biobank / The National Institute of Health and Welfare Helsinki, Finland

Essi Kaiharju THL Biobank / The National Institute of Health and Welfare Helsinki, Finland

Markku Laukkanen THL Biobank / The National Institute of Health and Welfare Helsinki, Finland

Elina Järvensivu THL Biobank / The National Institute of Health and Welfare Helsinki, Finland

Sini Lähteenmäki THL Biobank / The National Institute of Health and Welfare Helsinki, Finland

Lotta Männikkö THL Biobank / The National Institute of Health and Welfare Helsinki, Finland

Regis Wong THL Biobank / The National Institute of Health and Welfare Helsinki, Finland

Registry Data Operations

Minna Brunfeldt THL Biobank / The National Institute of Health and Welfare Helsinki, Finland

Hannele Mattsson THL Biobank / The National Institute of Health and Welfare Helsinki, Finland

Kati Kristiansson THL Biobank / The National Institute of Health and Welfare Helsinki, Finland

Susanna Lemmelä Institute for Molecular Medicine Finland, HiLIFE, University of Helsinki, Finland

Sami Koskelainen THL Biobank / The National Institute of Health and Welfare Helsinki, Finland

Tero Hiekkalinna THL Biobank / The National Institute of Health and Welfare Helsinki, Finland

Teemu Paajanen THL Biobank / The National Institute of Health and Welfare Helsinki, Finland

Sequencing Informatics

Priit Palta Institute for Molecular Medicine Finland, HiLIFE, University of Helsinki, Finland

Kalle Pärn	Institute for Molecular Medicine Finland, HiLIFE, University of Helsinki, Finland
Mart Kals	Institute for Molecular Medicine Finland, HiLIFE, University of Helsinki, Finland
Shuang Luo	Institute for Molecular Medicine Finland, HiLIFE, University of Helsinki, Finland
Vishal Sinha	Institute for Molecular Medicine Finland, HiLIFE, University of Helsinki, Finland

Trajectory

Tarja Laitinen	Pirkanmaa Hospital District, Tampere, Finland
Mary Pat Reeve	Institute for Molecular Medicine Finland, HiLIFE, University of Helsinki, Finland
Marianna Niemi	University of Tampere, Tampere, Finland
Harri Siirtola	University of Tampere, Tampere, Finland
Javier Gracia-Tabuenca	University of Tampere, Tampere, Finland
Mika Helminen	University of Tampere, Tampere, Finland
Tiina Luukkaala	University of Tampere, Tampere, Finland
Iida Vähätalo	University of Tampere, Tampere, Finland

Data protection officer

Jyrki Pitkänen	Institute for Molecular Medicine Finland, HiLIFE, University of Helsinki, Finland

FINBB - Finnish biobank cooperative

Marco Hautalahti

Johanna Mäkelä

Sarah Smith

Tom Southerington
