# Supplementary material for: Genetic risk factors have a substantial impact on healthy life years
Source: Nat Med. 2022 Sep 12;28(9):1893–901. doi: 10.1038/s41591-022-01957-2 (PMC9499866; doi:10.1038/s41591-022-01957-2)
Supplement: Supplementary file 2 — Reporting Summary [file 41591_2022_1957_MOESM2_ESM.pdf]

nature portfolio

Corresponding author(s): Sakari Jukarainen, Andrea Ganna

Last updated by author(s): Jul 14, 2022

Reporting Summary

Nature Portfolio wishes to improve the reproducibility of the work that we publish. This form provides structure for consistency and transparency in reporting. For further information on Nature Portfolio policies, see our Editorial Policies and the Editorial Policy Checklist.

Statistics

For all statistical analyses, confirm that the following items are present in the figure legend, table legend, main text, or Methods section.

n/a	Confirmed	
☐	☒	The exact sample size (*n*) for each experimental group/condition, given as a discrete number and unit of measurement
☒	☐	A statement on whether measurements were taken from distinct samples or whether the same sample was measured repeatedly
☐	☒	The statistical test(s) used AND whether they are one- or two-sided<br>*Only common tests should be described solely by name; describe more complex techniques in the Methods section.*
☐	☒	A description of all covariates tested
☐	☒	A description of any assumptions or corrections, such as tests of normality and adjustment for multiple comparisons
☐	☒	A full description of the statistical parameters including central tendency (e.g. means) or other basic estimates (e.g. regression coefficient) AND variation (e.g. standard deviation) or associated estimates of uncertainty (e.g. confidence intervals)
☐	☒	For null hypothesis testing, the test statistic (e.g. *F*, *t*, *r*) with confidence intervals, effect sizes, degrees of freedom and *P* value noted<br>*Give P values as exact values whenever suitable.*
☐	☒	For Bayesian analysis, information on the choice of priors and Markov chain Monte Carlo settings
☒	☐	For hierarchical and complex designs, identification of the appropriate level for tests and full reporting of outcomes
☐	☒	Estimates of effect sizes (e.g. Cohen's *d*, Pearson's *r*), indicating how they were calculated

Our web collection on statistics for biologists contains articles on many of the points above.

Software and code

Policy information about availability of computer code

Data collection	No software was used for data collection in this study.
Data analysis	Software: R versions v4.0.3 and v4.1.2, PLINK v1.90 and v2.0 , Eagle v2.3.527, Beagle v4.128, KING v2.2, PRS-CS, IMPUTE4, SISu v3, VEP v103, hail v0.2, SAIGE v0.35.8.8, LDSC v1.0.1<br>R packages: tidyverse 1.3.1, susieR v0.8.1, survival v3.2-11, coxphf v1.13.1, data.table v1.14.2, ggrepel v0.9.1<br>Code for central parts of analyses can be found at https://github.com/dsgelab/dalys_code

For manuscripts utilizing custom algorithms or software that are central to the research but not yet described in published literature, software must be made available to editors and reviewers. We strongly encourage code deposition in a community repository (e.g. GitHub). See the Nature Portfolio guidelines for submitting code & software for further information.

Data

Policy information about availability of data

All manuscripts must include a data availability statement. This statement should provide the following information, where applicable:

- Accession codes, unique identifiers, or web links for publicly available datasets
- A description of any restrictions on data availability
- For clinical datasets or third party data, please ensure that the statement adheres to our policy

We present meta-analyzed HR estimates and all attributable DALY results in Supplementary Tables 5-6, 8-9, and ST11-14. Results for common variants, HLA alleles, PGSes can additionally be explored through plots at https://dsge-lab.shinyapps.io/daly_genetics/. Individual-level genotypes and register data from FinnGen participants can be accessed by approved researchers via the Fingenious portal (https://site.fingenious.fi/en/) hosted by the Finnish Biobank Cooperative FinBB

(https://finbb.fi/en/). Data release to FinBB is timed to the bi-annual public release of FinnGen summary results which occurs twelve months after FinnGen consortium members can start working with the data.
UK Biobank data is available to approved researchers via application (https://www.ukbiobank.ac.uk/enable-your-research/apply-for-access).
Some of the datasets used in this study can be accessed in: ClinVar (https://www.ncbi.nlm.nih.gov/clinvar/) and gnomAD (https://gnomad.broadinstitute.org/).

Field-specific reporting

Please select the one below that is the best fit for your research. If you are not sure, read the appropriate sections before making your selection.

☒ Life sciences ☐ Behavioural & social sciences ☐ Ecological, evolutionary & environmental sciences

For a reference copy of the document with all sections, see nature.com/documents/nr-reporting-summary-flat.pdf

Life sciences study design

All studies must disclose on these points even when the disclosure is negative.

Sample size	Sample size was determined by available data.
Data exclusions	Individuals of non-Finnish ancestry in FinnGen were excluded via ancestry analysis. Similarly in UK Biobank individuals of non-European ancestry were excluded (see Online Methods for details).
Replication	There was no attempt to replicate the results.
Randomization	Not relevant, observational study.
Blinding	Not relevant, observational study.

Reporting for specific materials, systems and methods

We require information from authors about some types of materials, experimental systems and methods used in many studies. Here, indicate whether each material, system or method listed is relevant to your study. If you are not sure if a list item applies to your research, read the appropriate section before selecting a response.

Materials & experimental systems

n/a	Involved in the study	
☒	☐	Antibodies
☒	☐	Eukaryotic cell lines
☒	☐	Palaeontology and archaeology
☒	☐	Animals and other organisms
☐	☒	Human research participants
☒	☐	Clinical data
☒	☐	Dual use research of concern

Methods

n/a	Involved in the study	
☒	☐	ChIP-seq
☒	☐	Flow cytometry
☒	☐	MRI-based neuroimaging

Human research participants

Policy information about studies involving human research participants

Population characteristics	For FinnGen see https://doi.org/10.1101/2022.03.03.22271360 for UK Biobank see https://pubmed.ncbi.nlm.nih.gov/25826379/<br><br>Sex was considered in the analyses by stratifying individuals by sex for some analyses. Sex was determined from genotyping analysis as part of the quality control procedures for FinnGen and UK Biobank.
Recruitment	For FinnGen see https://doi.org/10.1101/2022.03.03.22271360 for UK Biobank see https://pubmed.ncbi.nlm.nih.gov/25826379/
Ethics oversight	The FinnGen study is approved by Finnish Institute for Health and Welfare (permit numbers: THL/2031/6.02.00/2017, THL/1101/5.05.00/2017, THL/341/6.02.00/2018, THL/2222/6.02.00/2018, THL/283/6.02.00/2019, THL/1721/5.05.00/2019 and THL/1524/5.05.00/2020), Digital and population data service agency (permit numbers: VRK43431/2017-3, VRK/6909/2018-3, VRK/4415/2019-3), the Social Insurance Institution (permit numbers: KELA 58/522/2017, KELA 131/522/2018, KELA 70/522/2019, KELA 98/522/2019, KELA 134/522/2019, KELA 138/522/2019, KELA 2/522/2020, KELA 16/522/2020), Findata permit numbers THL/2364/14.02/2020, THL/4055/14.06.00/2020,,THL/3433/14.06.00/2020, THL/4432/14.06/2020, THL/5189/14.06/2020, THL/5894/14.06.00/2020, THL/6619/14.06.00/2020, THL/209/14.06.00/2021, THL/688/14.06.00/2021, THL/1284/14.06.00/2021, THL/1965/14.06.00/2021, THL/5546/14.02.00/2020 and Statistics Finland (permit numbers: TK-53-1041-17 and TK/143/07.03.00/2020 (earlier TK-53-90-20)).

UK Biobank study has obtained ethics approval from the North West Multi-centre Research Ethics Committee which covers the UK (approval number: 11/NW/0382) and has obtained informed consent from all participants.

Note that full information on the approval of the study protocol must also be provided in the manuscript.
